# Nonadiabatic Molecular Dynamics Simulations Provide Evidence for Coexistence of Planar and Nonplanar Intramolecular Charge Transfer Structures in Fluorazene

**DOI:** 10.1021/acs.jpca.4c03693

**Published:** 2024-08-07

**Authors:** Michał Andrzej Kochman

**Affiliations:** † Institute of Physical Chemistry of the Polish Academy of Sciences, Ul. Marcina Kasprzaka 44/52, 01-224 Warsaw, Poland; ‡ Theoretical Chemistry, Ruhr University Bochum, Universitätsstraße 150, 44801 Bochum, Germany

## Abstract

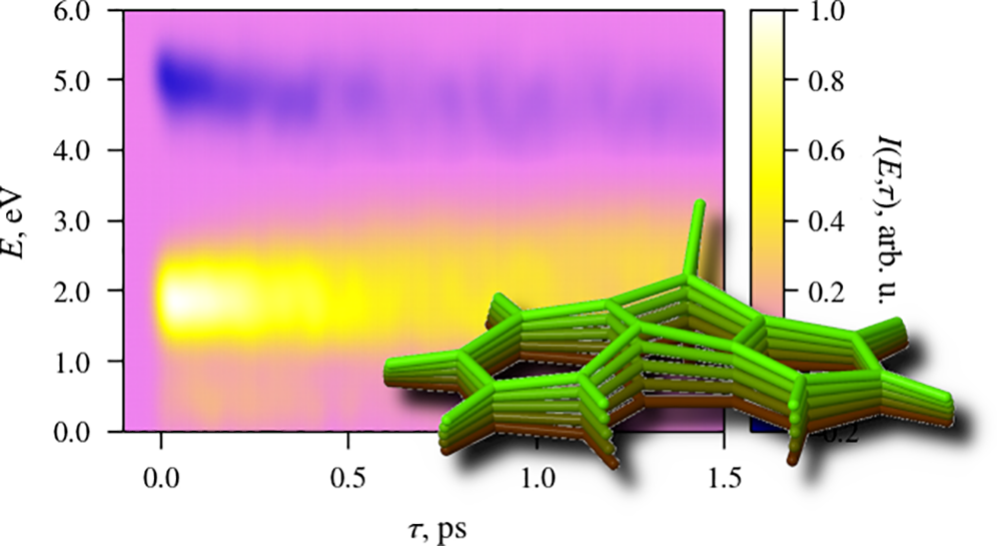

Fluorazene is a model compound for photoinduced intramolecular charge transfer (ICT) between aromatic moieties. Despite intensive studies, both spectroscopic and theoretical, a complete model of its photophysics is still lacking. Especially controversial is the geometry of its ICT structure, or structures. In order to fill in the gaps in the state of knowledge on this important model system, in the present study I report the results of nonadiabatic molecular dynamics (NAMD) simulations of its photorelaxation process in acetonitrile solution. To afford a direct comparison to spectroscopic data, I use the simulation results as the basis for the calculation of the transient absorption (TA) spectrum. The NAMD simulations provide detailed information on the sequence of events during the excited-state relaxation of the title compound. Following initial photoexcitation into the bright S_2_ state, the molecule undergoes rapid internal conversion into the S_1_ state, leading to the locally excited (LE) structure. The LE structure, in turn, undergoes isomerization into a population of ICT structures, with geometries ranging from near-planar to markedly nonplanar. The LE → ICT isomerization reaction is accompanied by the decay of the characteristic excited-state absorption band of the LE structure near 2 eV. The anomalous fluorescence emission band of fluorazene is found to originate mainly from the near-planar ICT structures, in part because they dominate the overall population of ICT structures. Thus, the planar ICT (PICT) model appears to be the most appropriate description of the geometry of the ICT structure of fluorazene.

## 1 Background

**Figure 1:**
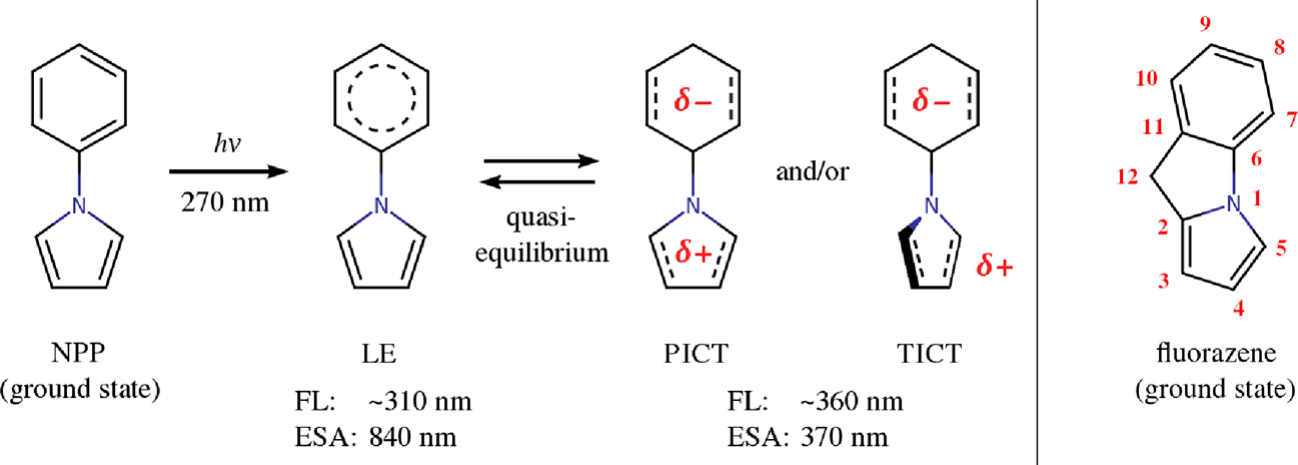
(a) Molecular structure and photophysics of *N*-phenylpyrrole (NPP). “FL” denotes the position of the fluorescence emission band of either excited-state structure. “ESA” is the position of the most intense excited-state absorption band of either structure. (b) Molecular structure of fluorazene, the rigidized counterpart of NPP.

Photoinduced charge transfer between aromatic moieties plays a crucial role in several technologies, including organic photovoltaics^1−4^ and catalysts for photoinduced water splitting.^5,6^ Despite widespread interest in charge transfer states of this type, many aspects of their structure and reactivity remain incompletely understood. A case in point is the photophysics of *N*-phenylpyrroles^7^ (also known as 1-phenylpyrroles), a class of donor–acceptor compounds known precisely as model systems for intramolecular charge transfer (ICT) between aromatic groups. Figure 1 (a) shows a summary of the available spectroscopic data on the namesake compound *N*-phenylpyrrole (NPP), which is representative of *N*-phenylpyrroles generally.

In the ground electronic state, the geometry of the NPP molecule is close to planar–the pyrrole moiety is only slightly skewed with respect to the phenyl moiety.^8^ The molecule is essentially nonpolar, being characterized by a small electric dipole moment of 1.39 D (debye).^9^

At a short time delay (on the order of 0.5 ps) after excitation in the ultraviolet, NPP shows a single fluorescence band at around 310 nm,^10^ which is conventionally termed the normal band. It arises from the locally excited (LE) structure, so-called because of its small electric dipole moment of around 2 D, whose direction is approximately opposite to the dipole moment of the ground state.^11^ In polar organic solvents such as alkyl nitriles, the intensity of the 310 nm band decreases on a time scale of picoseconds, while simultaneously a second fluorescence band appears at around 360 nm.^10^ This latter band is termed the “anomalous” band, and it is attributed to one or more highly polar intramolecular charge transfer (ICT) structures, with an electric dipole moment of roughly 13 D.^11^ In the language of theoretical chemistry, the LE and the ICT structures correspond to minima on the potential energy surface (PES) of state S_1_, which differ from one another in terms of electronic structure. The appearance of two fluorescence bands originating from the same fluorophore is conventionally termed dual fluorescence.

Eventually, the LE and the ICT structures reach a quasi-equilibrium, and the emission profile stabilizes. (The two–or more–excited-state structures interconvert between one another, but their coexistence is not, strictly speaking, an instance of chemical equilibrium, because they are continually being depopulated through radiative and nonradiative decay processes.) The intensity ratio of the anomalous band to the normal band increases with increasing solvent polarity.^10^

There has been some debate over whether the isomerization between the LE and the ICT structures of NPP and related compounds is accompanied by a large-amplitude deformation of the heavy-atom skeleton. There is a consensus that the LE structures of *N*-phenylpyrroles are characterized by planar or near-planar geometries, a view that is uniformly supported by theoretical studies. More controversial are the geometries of the ICT structures. In the early theoretical studies of NPP by Parusel^12^ and by Proppe and co-workers,^13^ the anomalous fluorescence band was ascribed to a twisted ICT (TICT) structure with a perpendicular orientation of the pyrrole and the phenyl moieties.

Yoshihara and co-workers^14^ undertook to verify the hypothesis that the anomalous fluorescence of *N*-phenylpyrroles arises from TICT structures. To that end, these authors investigated the photophysics of fluorazene (see Figure 1 (b)), a derivative of NPP in which the relative orientation of the pyrrole and the phenyl moieties is constrained by a methylene linker. (The nonsystematic name “fluorazene” was apparently coined by Laschtuvka and Huisgen.^15^) Tethering the electron-donating and -accepting moieties was by then a well-established strategy for the study of the geometries of ICT states.^16−18^

In the event, Yoshihara et al. found that the photophysics of fluorazene is largely similar to that of NPP. In particular, fluorazene does also exhibit dual fluorescence in polar solvents; the maximum of its anomalous fluorescence band is slightly red-shifted with respect to NPP.^14^ What is more, it was determined that the LE → ICT reaction is faster by a factor of 2 for fluorazene than for NPP under the same conditions.^14^

Since fluorazene is incapable of undergoing significant intramolecular rotation, it follows that a twisting of the pyrrole moiety is not a prerequisite for the occurrence of ICT, and for dual fluorescence. In fact, Yoshihara et al. went further than that, and concluded that the ICT structures of fluorazene as well as other *N*-phenylpyrroles are planar.^14^ This model of the geometry of the ICT structure is usually denoted as PICT.

Complicating the picture, subsequent theoretical studies by Xu et al.^19^ and by Galván et al.^20^ have indicated that fluorazene can potentially adopt two ICT structures, of which one is planar (PICT-type), and the other is markedly nonplanar (“bent”) with a pyramidalized carbon atom C6 (see Figure 3 later on in this paper). The possible existence of the bent ICT structure implies that the occurrence of dual fluorescence in fluorazene is not necessarily an argument for the planarity of the ICT structures of *N*-phenylpyrroles. The photoexcited fluorazene molecule does retain some flexibility, despite the partial rigidization of the heavy-atom skeleton by the methylene linker.

Unfortunately, it remains unclear which ICT structure makes the dominant contribution to the anomalous fluorescence band of any given compound. In this regard, Xu et al. attributed the anomalous band of fluorazene to the bent ICT structure.^19^ Conversely, Galván et al.^20^ argued that calculated fluorescence emission energy of the bent ICT structure is too low to correspond to the anomalous fluorescence band, and that the band in question instead originates from the PICT structure. At the same time, however, Galván et al.^20^ were careful to point out that assigning anomalous fluorescence band to the PICT structure leaves open the question of why the bent ICT structure does not give rise to a separate fluorescence band at a lower energy.^20^

As a side note, the situation with *N*-phenylpyrroles is superficially similar to the debate over the photophysics of aminobenzonitriles,^21^ another well-known class of compounds which exhibit dual fluorescence. There is an important difference between the two types of fluorophore, though: in the ICT structures of aminobenzonitriles, the charge donor is the amino group nitrogen.^22^ Conversely, in the ICT structures of *N*-phenylpyrroles, it is the C=C bonds of the pyrrole moiety that act as the main charge donors.^20,23^ Furthermore, aminobenzonitriles and *N*-phenylpyrroles respond differently to constraints on the relative motion of the electron-donating and -accepting moieties.^16−18^ As a consequence, it cannot be assumed that the geometries of the ICT states of *N*-phenylpyrroles are similar to those of aminobenzonitriles.

Motivated by the long-standing controversy surrounding the photophysics of NPP and its derivatives, in the present contribution I report nonadiabatic molecular dynamics (NAMD) simulations of the excited-state relaxation dynamics of fluorazene in acetonitrile (ACN). The reason I selected fluorazene as the system for study is that the partially rigidized molecular skeleton makes it an especially interesting problem for theoretical investigation. As mentioned above, the LE → ICT isomerization reaction is known to be faster in fluorazene than in NPP.^14^ I therefore expected that choosing fluorazene over NPP as the model system would improve the prospects of observing the LE → ICT reaction within the finite time scale of the NAMD simulation.

Next to fluorescence spectroscopy, transient absorption (TA) spectroscopy^24−27^ has played a major role in elucidating the relaxation dynamics of fluorazene and other *N*-phenylpyrroles in the solution phase. In order to tie in the simulation results with the available spectroscopic data, I used the results of the NAMD simulations as the basis for the calculation of the transient absorption (TA) spectrum.

The rest of the paper is organized as follows. In the following section, I describe the simulation setup, with special regard to the level of realism achieved by my methodology. I then move on to describe the sequence of events in the photorelaxation dynamics of fluorazene. Finally, I analyze the simulated TA spectrum of fluorazene in light of the available experimental data.

## 2 Computational Methods

### 2.1 NAMD Simulations

The aim of the NAMD simulations was to model the relaxation dynamics of fluorazene in ACN solution following photoexcitation in the UV range, and thereby to provide the basis of the simulation of the TA spectrum. The computational methodology was adapted from the approach used previously by Kochman and co-workers^28,29^ in simulation studies of another archetypal donor–acceptor compound, 4-(*N*,*N*-dimethylamino)benzonitrile (DMABN). It is also worth referencing here the recent study by Avagliano and co-workers, who used DMABN in ACN as the test system for an automated workflow for the modeling of TA spectra.^30^ The dilute solution phase was represented by placing a single fluorazene molecule at the center of a spherical nanodroplet consisting of 500 ACN molecules. The PESs of the system were constructed with the use of the additive variant of the quantum mechanics/molecular mechanics (QM/MM) method.^31−35^ In this approach, the system under study (denoted ) is partitioned into two subsystems which are treated at different levels of approximation. The electronic structure of the inner subsystem () is described explicitly with the use of a quantum mechanical (QM) method. The outer subsystem (), in turn, is described with a molecular mechanics (MM) force field.

**Figure 2:**
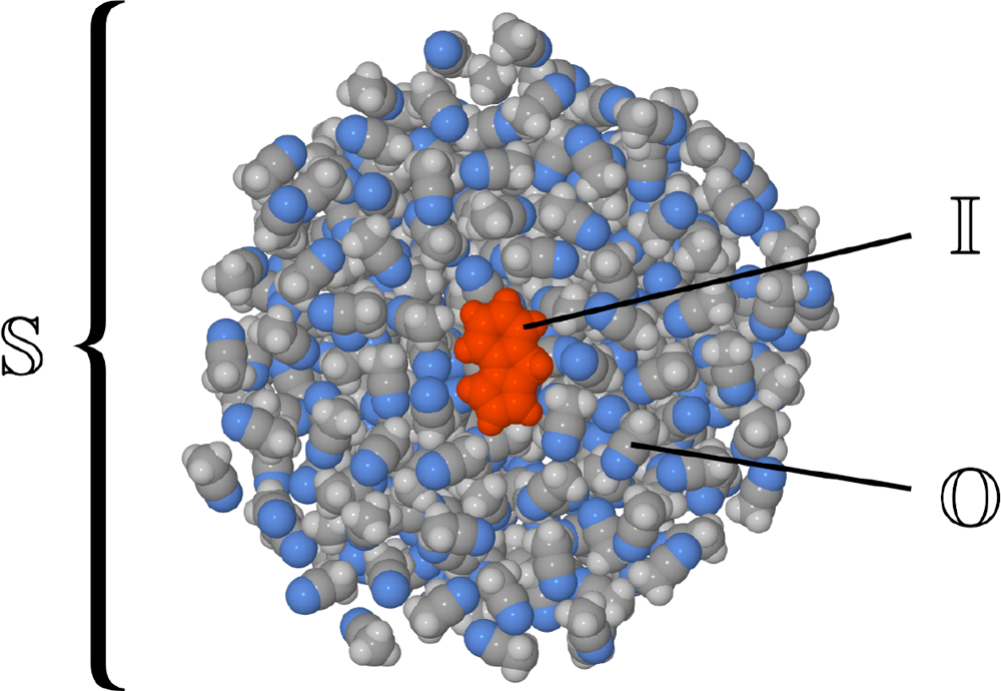
Schematic illustration of the partitioning of the system (denoted ) into the inner () and outer () subsystems. The figure shows a cross section of the 500-molecule ACN droplet enclosing the fluorazene molecule. In the actual simulations, the solute molecule is surrounded by the solvent on all sides.

As shown in Figure 2, in the present case the inner subsystem consisted of the fluorazene molecule, and the ACN molecules collectively comprised the outer subsystem. The electronic structure of fluorazene was described with time-dependent density functional theory^36−39^ (TDDFT). The solvent was described with the all-atom optimized potentials for liquid simulations^40,41^ (OPLS-AA) force field. The polarization of the fluorazene molecule by the solvent, which plays a crucial role in the dual fluorescence process, was accounted for by including the point charges of the solvent in the QM Hamiltonian. This treatment of electrostatic interactions between the inner and the outer subsystems is usually referred to as electrostatic embedding.^32^

The initial conditions for the NAMD simulations were generated in such a way as to represent photoexcitation by a pump pulse, whose energy range was set to 5.1 ± 0.1 eV. As per standard practice in NAMD simulations of photorelaxation processes in the condensed phase,^42,43^ when generating the initial conditions, the solute and the solvent were treated on an unequal footing. Namely, the initial positions and velocities of the solute atoms were sampled from the Wigner distribution. The solvent configurations, in turn, were sampled from thermostatted molecular dynamics simulations in which the geometry of the solute was frozen.

The subsequent relaxation dynamics was simulated with the fewest switches surface hopping (FSSH) algorithm.^44−47^ A set of *N*_trajs_ = 60 trajectories were propagated for a period of 1.5 ps. The electronic wave function of the fluorazene molecule was expressed as a linear combination of states S_1_ to S_4_. Internal conversion to the ground state (S_0_) was not taken into account, as the excited-state lifetime of fluorazene is on the order of nanoseconds,^14,48^ 3 orders of magnitude longer than the time scale of the present simulations. It is well documented that the basic FSSH algorithm does not account for decoherence effects.^49−51^ Accordingly, during the NAMD simulations, the state coefficients were corrected for decoherence via the energy-based scheme proposed by Granucci and Persico.^47^ A more detailed description of the simulation setup, including the rationale for the choice of some of the adjustable parameters, is provided in Section S2 of the Supporting Information (SI).

The following three parameters were used to monitor the relaxation dynamics. The electronic state of the fluorazene molecule in the ensemble of simulated trajectories was tracked by calculating the classical populations of states S_1_ to S_4_. As per the usual convention, the classical population (*P*_*j*_) of the *j*th state is defined as the fraction of trajectories that are currently occupying that state:
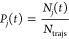
1

It is also of interest to quantify the degree of charge separation in the occupied electronic state along each trajectory. To this end, I monitored the magnitude of the mean hole–electron separation vector (|*d⃗*_h→e_|) as defined by Plasser and co-workers.^52^

As noted in the Background section, one of the possible ICT structures of fluorazene is characterized by the pyramidalization of atom C6 (see Figure 1 for atom numbering), which causes the molecule as a whole to adopt a markedly nonplanar geometry. Accordingly, I also tracked the deviation of the heavy-atom skeleton from planarity. This was done as follows. A plane *p* was fitted to the positions of the heavy atoms of the fluorazene molecule in such a way as to minimize the root-mean-square distance (RMSD) between the plane and the positions of the heavy atoms:
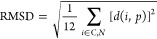
2

Here, *d*(*i*, *p*) is the distance between the *i*th atom and plane *p*, measured perpendicular to plane *p*. The factor of 1/12 comes from the fact that there are 12 heavy atoms in the fluorazene molecule. The RMSD value remaining after minimization (denoted RMSD_min_) was taken as a measure of the planarity of the molecular skeleton. The fitting was performed anew whenever parameter RMSD_min_ was calculated, owing to which the value of RMSD_min_ is invariant to the translation and rotation of the molecule.

**Figure 3:**
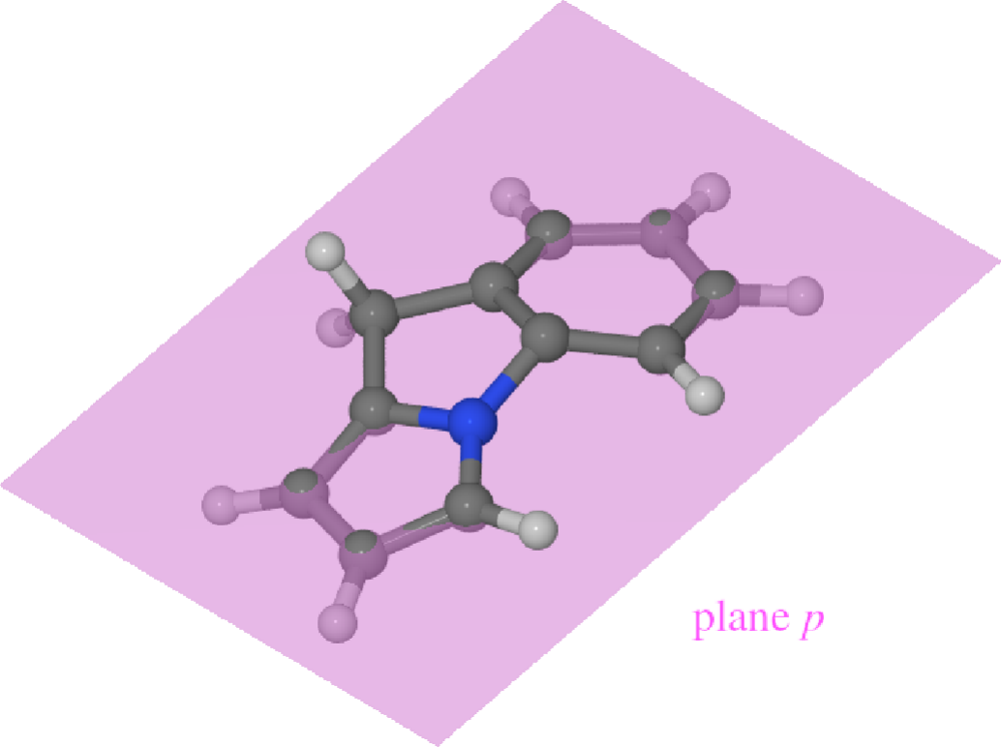
Illustration of parameter RMSD_min_. Plane *p* has been drawn semitransparent.

As an illustration of the functioning of parameter RMSD_min_ in practice, Figure 3 shows the plane fitted to the bent ICT structure of fluorazene as optimized at the TDDFT level of theory (see the following section for the computational parameters). It can be seen that atoms C6, C7, C11, and N1 are displaced above plane *p*, and they will contribute substantially to the value of RMSD_min_. As a rule of thumb, the molecular geometry can be said to be markedly bent if RMSD_min_ takes a value of around 0.2 Å or higher. This criterion is based on the fact the bent ICT minimum-energy geometry optimized at the TD-CAM-B3LYP/def2-SVP level has RMSD_min_ = 0.23 Å.

### 2.2 Calculation of TA Spectrum

Much of what is known about the photophysics of fluorazene and other *N*-phenylpyrroles in the solution phase comes from the series of TA spectroscopic studies by the group of Zachariasse.^10,14,48,53^ In order to enable a direct comparison of simulation results to experimental data, I undertook to model the TA spectrum of the simulated system. More formally, I evaluated the difference spectrum *I*(*E*, τ), which is given by the absorption spectrum of the photoexcited sample at probe pulse photon energy *E* and delay time τ, minus the absorption spectrum of the sample in the ground state. Under some simplifying approximations, the signal intensity in the difference spectrum is proportional to the superposition of the excited-state absorption (ESA) and stimulated emission (SE) contributions from the individual NAMD trajectories:^54^
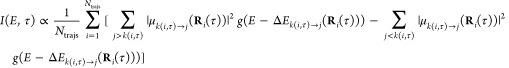
3

**Figure 4:**
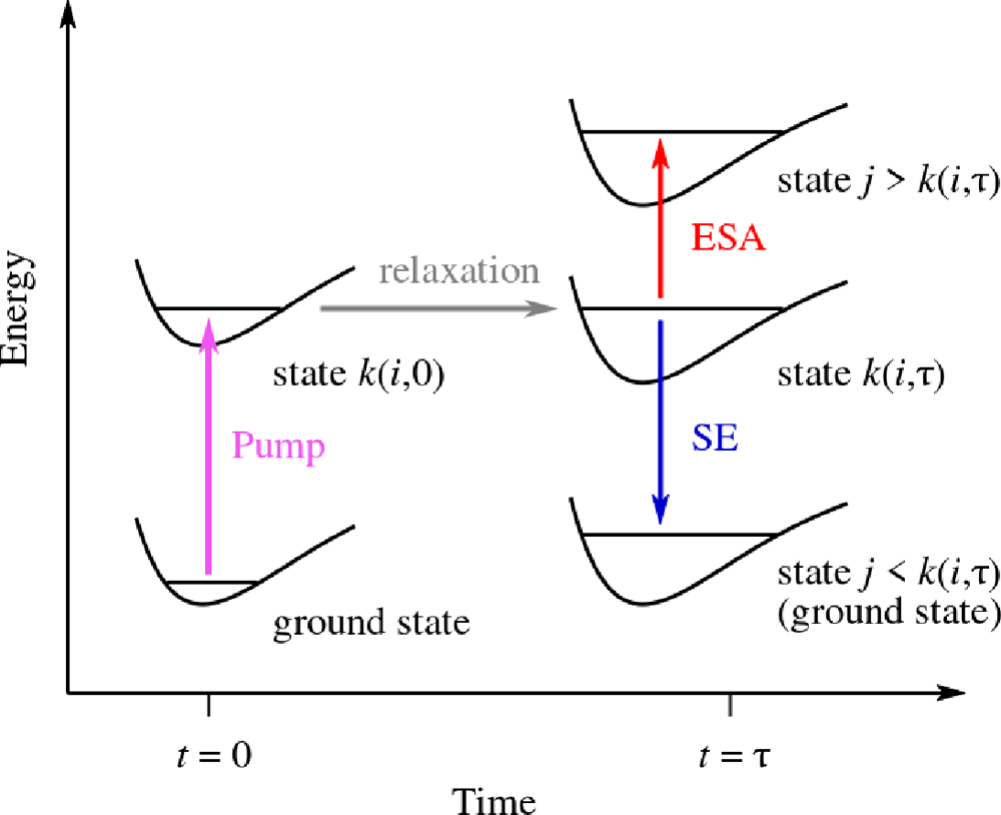
Calculation of a TA spectrum on the basis of NAMD simulations. τ is the delay time between the pump pulse and the probe pulse. *k*(*i*, *t*) is the occupied state at time *t* in the *i*th NAMD trajectory.

For the sake of clarity, some of the quantities which appear in eq 3 are defined visually in Figure 4. The first inner sum is the contribution from ESA, and it involves states *j* which lie higher in energy than the occupied state, *k*(*i*, *t*). The second inner sum is the contribution from SE, and it involves states *j* which lie lower in energy than *k*(*i*, *t*), down to and including the ground state. μ_*k*(*i*,*t*)→*j*_(**R**_*i*_(*t*)) is the transition dipole moment for the transition from state *k*(*i*, *t*) to state *j* at geometry **R**_*i*_(*t*), and Δ*E*_*k*(*i*,*t*)→*j*_(**R**_*i*_(*t*)) is the energy of that transition. Lastly, *g*(*E* – Δ*E*_*k*(*i*,*t*)→*j*_(**R**_*i*_(*t*))) is a line shape function that is centered at Δ*E*_*k*(*i*,*t*)→*j*_(**R**_*i*_(*t*)). In the present study, I used a Gaussian line shape function with a standard deviation of 0.2 eV.

For the sake of simplicity, the dependence of signal intensities on the angle between the pump pulse and the probe pulse is neglected in expression 3. Disregarding this effect is also justified on the grounds that the TDDFT method is found to overestimate the intensity of ESA near 2 eV, such that the simulation only achieves semiquantitative accuracy in any case. Furthermore, I do not take into account the contribution from ground-state bleach (GSB). This is because the GSB signal falls outside the spectral range in which the difference spectrum of fluorazene was measured in ref (48), meaning that it does not need to be included in the simulations.

During the calculation of the spectrum, the signal intensity was evaluated at intervals of 20 fs. The sum over states for ESA which appears in eq 3 was truncated after state S_16_. In order to account for the finite time resolution of the experimentally measured spectrum, the raw simulated spectrum was subjected to a Gaussian blur in the time domain with a standard deviation of 20 fs.

### 2.3 Electronic Structure Calculations

My method of choice for the description the electronic structure of fluorazene is the combination of density functional theory (DFT) for the ground state with linear response TDDFT for the excited states. This choice of method is motivated by two factors. First, the emergence of the quasi-equilibrium between the LE and the ICT structures is a relatively slow process by the standards of NAMD simulations. This necessitates the use of a computationally efficient method. Second, propagating the dynamics of the system requires access to excited-state gradients and nonadiabatic coupling elements (or, alternatively, nonadiabatic coupling vectors) between excited states. TDDFT meets both these criteria.

Prior to the NAMD simulations, the performance of TDDFT for fluorazene was assessed by comparing it to the benchmark provided by more accurate wave function-based methods. The detailed discussion of these calculations is relegated to Section S1 of the SI. In brief, I determined that TDDFT applied with the CAM-B3LYP exchange-correlation functional^55^ provides a qualitatively correct description of the topography of the excited-state potential energy surfaces (PESs) of fluorazene. In quantitative terms, according to the benchmark provided by third-order algebraic-diagrammatic construction method^56,57^ (ADC(3)) method, the bent ICT structure is artificially stabilized relative to the LE structure by roughly 0.4 eV. When it comes to the calculation of the TA spectrum, it is found that the TD-CAM-B3LYP approach overestimates the intensity of at least some ESA transitions in the range of around 2–3 eV, but otherwise it performs reasonably well for ESA transitions with energies up to around 3 eV. At energies above around 3 eV, TD-CAM-B3LYP is no longer reliable. This level of accuracy is deemed acceptable for the purposes of this study.

For technical reasons (the availability of different type of calculations), the TDDFT part of the overall simulation was performed with two different programs: TURBOMOLE^58,59^ (version 7.5.0) was used for the calculation of PESs and excited-state gradients. Q-Chem^60,61^ (version 5.1.2) was employed to calculate all transition dipole moments, nonadiabatic coupling vectors between excited states, as well as the mean hole–electron separation vector. Insofar as possible, the same simulation settings were used for both programs: the CAM-B3LYP functional^55^ was employed in combination with the def2-SVP basis set.^62^ Cartesian d basis functions were used at all times. The Tamm–Dancoff approximation^63^ was imposed. The TURBOMOLE calculations (only) used the RI-*J* approximation^66^ with the default auxiliary basis set for def2-SVP,^67^ and in these calculations, I employed the fine m5 integration grid.^64^ In the Q-Chem calculations, I used the standard integration grid SG-1.^65^ I have verified that, with this choice of simulation parameters, the two programs give nearly identical results. In particular, excitation energies calculated at the TD-CAM-B3LYP/def2-SVP level coincide to within 0.001 eV. Thus, state energies and gradients obtained with TURBOMOLE can be used alongside NACVs calculated with Q-Chem.

## 3 Results and Discussion

### 3.1 Relaxation Dynamics

**Figure 5:**
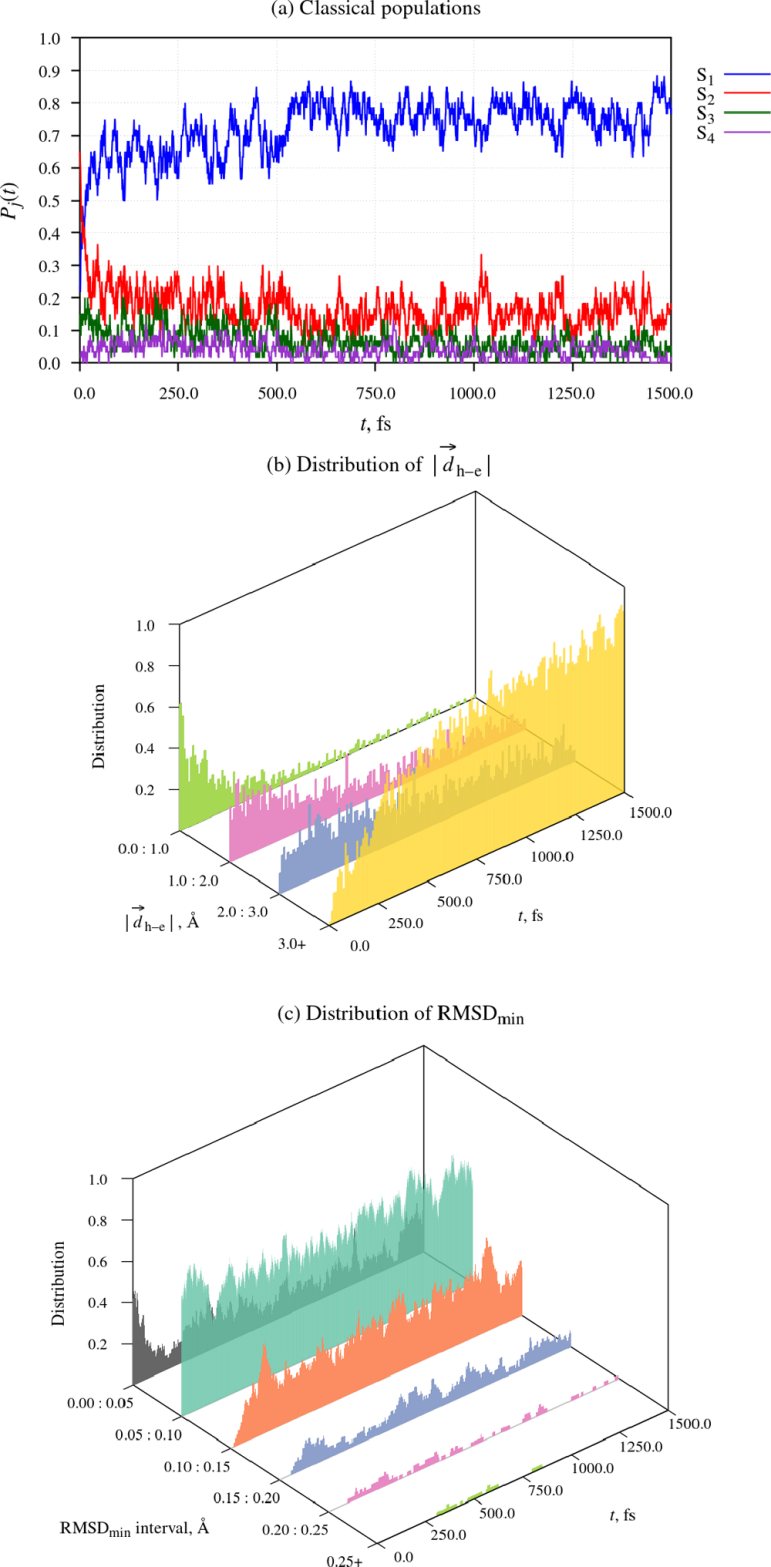
Time evolution of molecular geometry and electronic structure during the relaxation dynamics of fluorazene in the 500-molecule ACN nanodroplet. *t* = 0 corresponds to the initial photoexcitation by the pump pulse. In panels (b) and (c), selected histograms were drawn semitransparent, so that they do not obscure the other histograms.

I begin the discussion of the simulation results with an overview of the photorelaxation dynamics of fluorazene in the ACN nanodroplet. The time evolution of the molecular geometry and the electronic structure of the system is characterized in Figure 5. Panel (a) shows the classical populations of states S_1_ to S_4_. Panel (b), in turn, shows the distribution of the magnitude of the mean hole–electron separation vector (|*d⃗*_h→e_|). Lastly, panel (c) presents the distribution of parameter RMSD_min_. The latter two quantities are presented in the form of histograms plotted as functions of time.

At the outset of the simulations, the geometries of the fluorazene molecule in the simulated trajectories were clustered in a narrow region of configuration space around the planar ground-state equilibrium geometry. As can be seen in Figure 5 (a), the pump pulse mainly populated state S_2_, which makes the largest contribution to the first photoabsorption band of fluorazene. States S_1_ and S_3_ were populated to a smaller extent, while the initial population of state S_4_ was negligible (only a single trajectory out of 60 started out occupying that state).

The initial, roughly 50 fs-long period of time immediately following photoexcitation was marked by a rapid, albeit incomplete, internal conversion from state S_2_ to state S_1_. As discussed in more detail in Section S3 of the SI, this process is predominantly driven by the deformation modes (bond stretching and bending) of the six-membered ring, and the stretching of bonds N1–C6, N1–C2, and N1–C5.

The direct product of the early S_2_ → S_1_ internal conversion is the near-planar LE structure. Its identity can be confirmed by inspecting the distributions of parameters |*d⃗*_h→e_| and RMSD_min_ at around *t* = 50 fs. It can be seen that values of |*d⃗*_h→e_| at this point in time largely fell in the range of 0.0–2.0 Å, which indicates a slight to moderate degree of charge separation. Moreover, values of RMSD_min_ fell in the range of 0.00–0.10 Å, meaning that the heavy-atom skeleton of the molecule was close to planar at this time.

Over the next few hundred femtoseconds, the distribution of |*d⃗*_h→e_| gradually shifted toward higher values (3.0 Å and higher), a clear sign that the fluorazene molecule had undergone ICT. While it is difficult to pinpoint the exact onset of the LE → ICT reaction, it is apparent that, by *t* = 250 fs, there was already a substantial buildup of the ICT structures. From around *t* = 750 fs, the distribution was strongly dominated by intermediate to high |*d⃗*_h→e_| values.

It is informative to quantify the rate of the LE → ICT reaction. For the purposes of this analysis, let us say that the ICT structures are those whose |*d⃗*_h→e_| values exceed an arbitrarily chosen threshold of 3.0 Å. (At the DFT-optimized ground-state equilibrium geometry of fluorazene, the distance between the geometric centers of the phenyl ring and the pyrrole ring is 3.85 Å. In order to make my ad hoc definition more inclusive, I round down to the nearest whole ångström–hence, 3.0 Å.) A least-squares fit of an exponential function to the population of such structures gives a time constant of 0.33 ps. This is shorter by a factor of roughly 5 than the LE → ICT reaction time of 1.6 ps reported by Druzhinin and co-workers.^48^ The most likely reason why the simulations underestimate the reaction time is that the TD-CAM-B3LYP level of theory artificially stabilizes at least some of the ICT structures relative to the LE structure.

The distribution of parameter RMSD_min_ gives more insight into the characteristics of the ICT structures. As mentioned above, at the time of the initial photoexcitation, the fluorazene molecule was near-planar. During the initial 250 fs-long period of time following photoexcitation, the fraction of bent molecular geometries increased somewhat, but then stabilized, such that bent molecular geometries never formed the majority. It follows that during the latter part of the simulation (after around *t* = 750 fs), bent ICT structures coexisted with near-planar ICT structures. Visual inspection of the simulated trajectories suggests that the fluorazene molecule can readily interconvert between near-planar and bent geometries on a time scale of a few hundreds of femtoseconds. One might therefore speak of a population of ICT structures with different geometries, rather than bent and near-planar ICT structures as separate species.

The predominance of near-planar ICT geometries is somewhat surprising in view of the results of geometry optimizations for the isolated fluorazene molecule, which are reported in Section S1 of the SI. Namely, according to the TD-CAM-B3LYP level of theory, the isolated molecule only has a single ICT-type minimum on the PES of state S_1_, which is characterized by a distinctly bent geometry. Thus, at first sight, there is a discrepancy between the results of the NAMD simulations, and isolated-molecule calculations. In order to understand this issue, I performed another set of geometry optimizations in which solvent–solute interactions were accounted for by including three explicit ACN molecules.

The results of these additional calculations are discussed in Section S5 of the SI. The key outcome is that the degree to which the geometry of the ICT structure deviates from planarity is sensitive to the positions of the solvating ACN molecules. In some of the fluorazene–3ACN clusters, the ICT structure adopts a near-planar geometry, while in others, it remains markedly bent. This supports my contention that, in the bulk solution phase, there is a distribution of ICT structures with geometries ranging from near-planar to bent.

Complementing the above narrative, as part of the electronic SI I have included MP4 animations of eight representative simulated trajectories. Each animation shows the fluorazene molecule and its first solvation shell. (More specifically, the animation shows all solvent molecules whose carbon and/or nitrogen atoms approach the solute molecule to within 4.0 Å at least once during the simulation. The remaining ACN molecules are hidden from view, so as not to obscure the fluorazene molecule.) The panel on the bottom shows the adiabatic energies of states S_1_ to S_4_ relative to the ground state. The passage of time is indicated with a vertical black line moving along the time axis. The currently occupied state is marked with a black circle.

### 3.2 Simulated TA Spectrum

**Figure 6:**
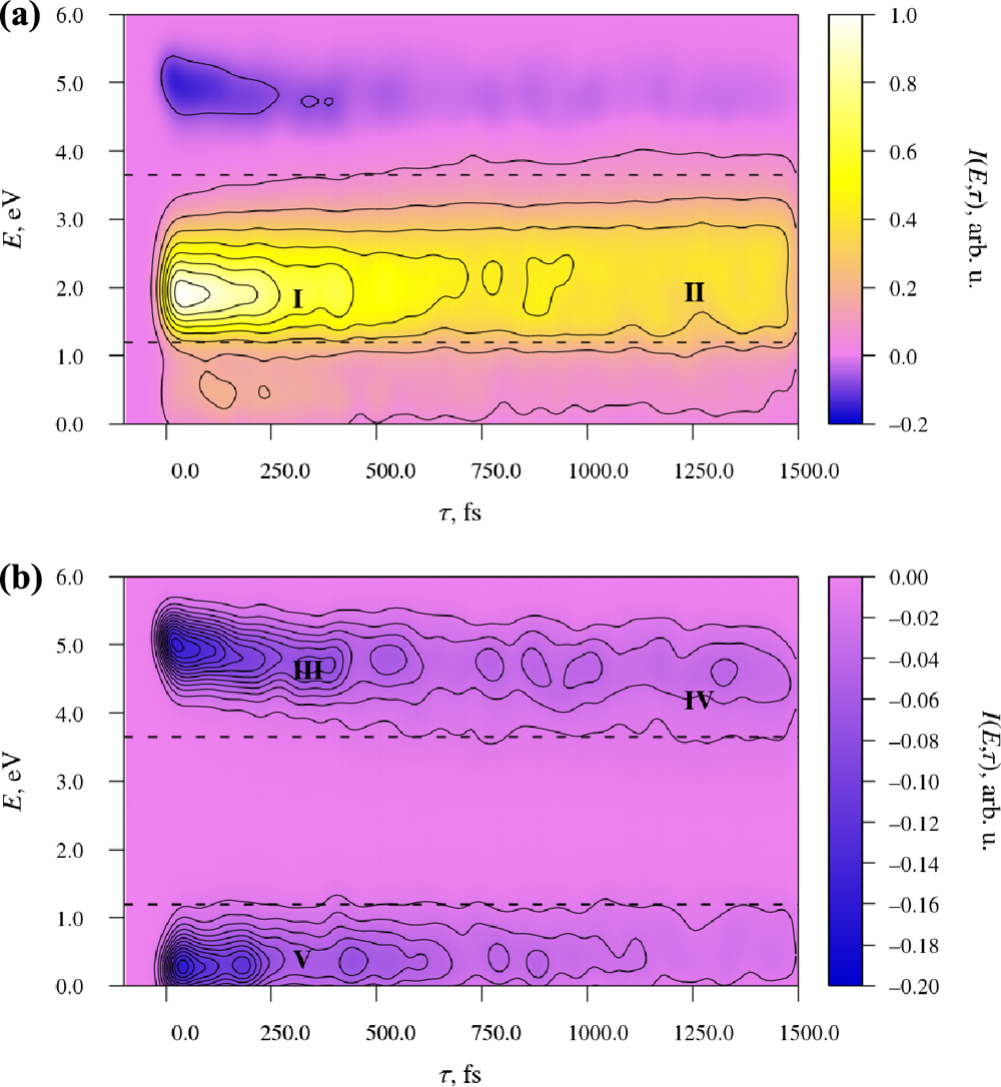
(a) Simulated TA spectrum of fluorazene in ACN. Signal intensity, in arbitrary units, is indicated with the use of color. The spectrum was normalized in such a way that the intensity maximum corresponds to a value of 1.0 on the intensity scale. Contour lines were overlaid on the color scale at intervals of 0.125. The dashed lines indicate the probe energy range of 1.19–3.65 eV which was used in ref (48). (b) SE contribution to the TA spectrum. Contour lines were overlaid on the color scale at intervals of 0.0125 (i.e., at finer intervals than in panel (a)).

Having examined the results of the NAMD simulations, I now move on to discuss the TA spectrum, which is plotted in Figure 6 (a). Note that, for the sake of completeness, I have plotted the entire photon energy range that is covered by the simulations, even though the reference spectroscopic data^48^ only covers the range of 1.19–3.65 eV (which corresponds to a wavelength range of 340–1040 nm).

At short pump–probe delay times of up to around τ = 500 fs, the TA spectrum shows an intense ESA signal in the energy range of around 1.5–2.1 eV. In Figure 6, this signal is labeled **I**. The 1.5–2.1 eV ESA signal matches the sharp, intense ESA signal seen in the experimentally measured TA spectrum of fluorazene in polar solvents^48^ in the energy range of around 1.5–1.8 eV (which corresponds to a wavelength range of roughly 850–700 nm). Its early appearance indicates that it originates from the LE structure, and its subsequent decay reflects the conversion of the LE structure into the ICT structures.

By *t* = 750 fs and later, the majority of simulated trajectories adopt ICT-type structures. These show relatively weak, unstructured ESA over a broad energy range of roughly 0.5–3.0 eV. In Figure 6 (a), this signal is labeled **II**.

The SE component of the TA spectrum is quite faint relative to the ESA component. In order to facilitate the analysis of the SE component, in Figure 6 (b) I plotted that component alone. At very short pump–probe delay times in the range of around 0–50 fs, the SE component shows a sharp, short-lived signal centered at an energy of 5.1 eV. From its short lifetime, I infer that this signal is mainly due to SE from state S_2_.

Immediately after the early S_2_ → S_1_ internal conversion process, the SE component shows a signal in the energy range of roughly 4.5–5.2 eV, which originates from the LE structure. In Figure 6 (b), this signal is labeled **III**. It gradually decays in intensity, giving way to a broad SE signal which spans the energy range of roughly 3.7–5.0 eV. This latter signal arises from the ICT structures, and it is marked **IV**.

Another SE signal is seen in the low energy range of around 0.0–1.2 eV In Figure 6 (b), this signal is marked **V**. It originates from S_*n*_ → S_1_ transitions (where *n* ≥ 2). This signal is not visible in the TA spectra reported in ref (48), as it falls almost completely outside the wavelength range in which the difference spectrum was measured.

The simulated TA spectrum can be compared directly to its experimentally observed counterpart. As mentioned already, the spectrum reproduces the appearance of the sharp, intense ESA signal of the LE structure of fluorazene, and its subsequent decay. The prediction that the ICT structures do not give rise to an ESA signal of comparable intensity is likewise in line with the experimental data.^48^ In this sense, the simulation correctly reproduces the main features of the observed spectrum.

Druzhinin et al.^48^ also reported that the LE structure exhibits a weak ESA band at around 2.9 eV (425 nm), while the ICT structure shows a weak ESA band at 3.4 eV (365 nm). These relatively less intense ESA signals are not reproduced by the simulations. A possible reason is that underlying transitions bring the molecule close to the ionization threshold, and they may therefore have a significant admixture of Rydberg character. Due to the small size of the def2-SVP basis set, the TDDFT calculations may be unable to correctly describe them.

The absence of the weak, high-energy ESA signals notwithstanding, the simulated TA spectrum is in reasonably good agreement with the experimental data. This suggests that the underlying NAMD simulations capture the main features of the relaxation dynamics of fluorazene in ACN.

### 3.3 Origin of Anomalous Fluorescence

**Figure 7:**
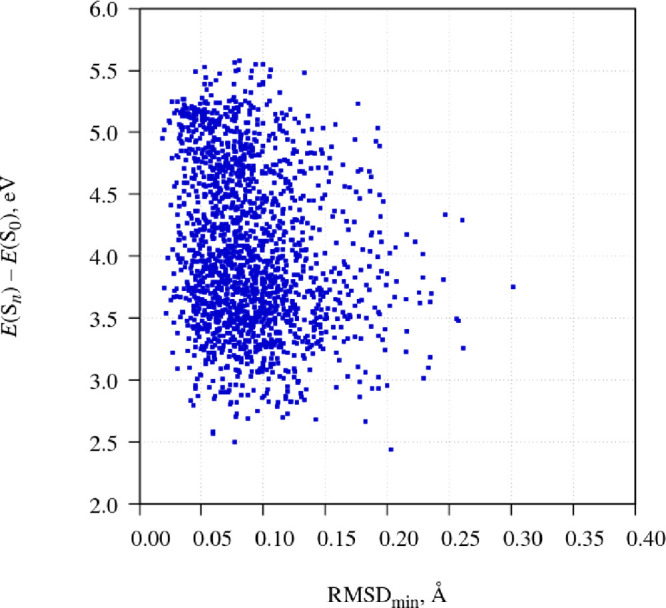
Scatter plot of the energy gap between the occupied state and the ground state versus parameter RMSD_min_.

My final order of business is to determine which ICT structure is responsible for the anomalous fluorescence band of fluorazene. In order to answer that question, in Figure 7 I generated a scatter plot of the energy gap between the occupied state (S_*n*_) and the ground state (S_0_) (on the vertical axis) versus parameter RMSD_min_ (on the horizontal axis). For the sake of clarity (to avoid crowding the plot with too many data points), I only included data points sampled from the simulated trajectories at intervals of 50 fs.

It turns out that the energy gap is not well correlated with the value of RMSD_min_. Data points with low values of the energy gap (which correspond to the anomalous fluorescence emission) are found both at low and at high values of RMSD_min_, though low values of RMSD_min_ (near-planar geometries) are far more numerous.

**Figure 8:**
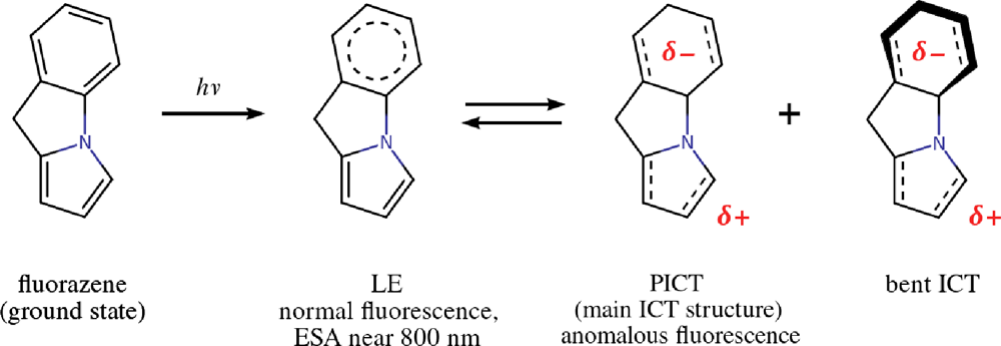
Schematic overview of the relaxation dynamics of fluorazene in ACN according to the present simulations.

The findings that the value of RMSD_min_ is a poor indicator of the energy gap, and near-planar geometries dominate in the population of ICT structures, suggest that the PICT model is the most appropriate description of the ICT structure of fluorazene. Bent ICT structures are also present in the ensemble of simulated trajectories, but they are in the minority. This situation is depicted schematically in Figure 8.

The question remains open whether the PICT model can be generalized to other *N*-phenylpyrroles. The simulation results obtained in the present study pertain strictly to the specific case of fluorazene, and they cannot be extrapolated to other compounds in this class. Future work is planned which will address the photophysics of NPP and other *N*-phenylpyrroles.

## 4 Conclusions

In this paper, I have constructed a comprehensive theoretical model of the photorelaxation dynamics of fluorazene, a rigidized donor–acceptor compound which has played a major role in the study of ICT between aromatic moieties. According to the mechanism which emerges from my simulations, internal conversion from the spectroscopically bright state S_2_ into state S_1_ occurs on a time scale of a few tens of femtoseconds, and the direct product of the internal conversion process is the near-planar LE structure. This structure gives rise to the characteristic ESA band near 2 eV.

The LE structure subsequently undergoes isomerization into a population of ICT structures with differing molecular geometries, ranging from near-planar to bent with a pyramidalized atom C6. The transition from the LE structure into the ICT structures is accompanied by a decay of the intense ESA band near 2 eV.

The simulation results indicate that there is little correlation between the bending of the molecular skeleton, and the energy gap between the occupied electronic state and the ground state. Given that the population of near-planar ICT structures is larger than the population of appreciably nonplanar structures, the PICT model, as proposed in refs (14 and 20), seems the most appropriate description of the structure of the ICT state of fluorazene. The caveat is that the PICT-type structures are predicted to coexist with a smaller fraction of markedly nonplanar (bent) ICT structures.

## Data Availability

In the interest of scientific reproducibility, the simulated NAMD trajectories have been made available for download at the Zenodo repository: https://doi.org/10.5281/zenodo.10966811 The NAMD simulations were performed with the use of a “wrapper” program, which acted as an interface to TURBOMOLE and Q-Chem. Its source code is available for download from the Zenodo repository: https://doi.org/10.5281/zenodo.12759064. Researchers interested in using this program are also encouraged to contact the author directly.

## Supporting Information Available

Benchmark calculations, setup of NAMD simulations, analysis of the origin of ultrafast S_2_ → S_1_ internal conversion, transition state for S_1_-LE–S_1_–ICT (BQ) isomerization, analysis of solvent–solute interactions in fluorazene–3ACN clusters, molecular geometries, and additional references (PDF)
Interpolated reaction pathway for S_1_-LE–S_1_–ICT (BQ) isomerization (ZIP)
Minimum-energy geometries of fluorazene–3ACN clusters (ZIP)
Animations of simulated trajectories (ZIP)
