# Supplementary material for: Nonadiabatic Molecular Dynamics Simulations Provide Evidence for Coexistence of Planar and Nonplanar Intramolecular Charge Transfer Structures in Fluorazene
Source: J Phys Chem A. 2024 Aug 7;128(32):6685–94. doi: 10.1021/acs.jpca.4c03693 (PMC11331525; doi:10.1021/acs.jpca.4c03693)
Supplement: Supplementary file 1 — jp4c03693_si_001.pdf [file jp4c03693_si_001.pdf]

Supporting Information for

Nonadiabatic Molecular Dynamics Simulations Provide Evidence for Coexistence of Planar and Non-Planar Intramolecular Charge Transfer Structures in Fluorazene

Michał Andrzej Kochman[†‡]

[†] Institute of Physical Chemistry, Polish Academy of Sciences, Ul. Marcina Kasprzaka 44/52, 01-224 Warsaw, Poland
[‡] Theoretical Chemistry, Ruhr University Bochum, Universitätsstraße 150, 44801 Bochum, Germany
e-mail: mkochman@ichf.edu.pl

Contents

S1 Benchmark Calculations **S2**
S1.1 Potential Energy Surfaces S2
S1.2 Excited-State Absorption Transitions S10

S2 Setup of NAMD Simulations **S15**
S2.1 QM/MM Calculations S15
S2.2 Initial Conditions for NAMD Simulations S17
S2.3 NAMD Algorithm S19

S3 Origin of Ultrafast $S_2 \rightarrow S_1$ Internal Conversion **S22**

S4 Transition State for S_1-LE–S_1-ICT (BQ) Isomerization **S23**

S5 Geometry of the Microsolvated ICT Structure **S24**

S6 Molecular Geometries **S29**
S_0-GS S29
S_1-LE S30
S_1-ICT (BQ) S31
S_1-TS S32

References **S33**

S1 Benchmark Calculations

As a preliminary to the simulation of the relaxation dynamics of fluorazene, I evaluated the accuracy of the chosen simulation methodology. The benchmark calculations were carried out over two stages. At the first stage, I verified whether the TD-CAM-B3LYP level of theory provides a realistic description of the excited-state potential energy surfaces (PESs) of fluorazene. At the second stage, I assessed the performance of this method in the calculation of the excited-state absorption (ESA) transitions.

S1.1 Potential Energy Surfaces

My first order of business was to examine the topography of the excited-state PESs of fluorazene predicted by the TD-CAM-B3LYP level of theory. To this end, I searched for, and optimized, minima on the PES of state S_1. For the sake of completeness, I also discuss here the ground-state equilibrium geometry as optimized at the density functional theory (DFT) level.

The benchmark calculations were performed for an isolated fluorazene molecule. The parameters of the DFT and the TDDFT calculations were the same as specified in Section 2.3 of the main body of this paper. In brief, the CAM-B3LYP functional[1] was employed in combination with the def2-SVP basis set.[2]

On the technical side, the geometry optimizations were performed by interfacing Q-Chem to the program Gaussian 16, Revision A.03.[3] In this setup, Gaussian acts as a “wrapper” around Q-Chem, and handles the geometry optimization by calling Q-Chem for the calculation of the energy and gradient. As per the default settings in Gaussian 16, the geometries were optimized with the use of the Berny algorithm in redundant internal coordinates.[4–11] No symmetry constraints were imposed. Each optimized geometry was confirmed to be a minimum on the PES of the given electronic state through a numerical calculation of vibrational frequencies.

The resulting ground- and excited-state minimum-energy geometries are characterized in Figure S2. For each geometry, I specify the value of parameter $\mathrm{RMSD_{min}}$, which was introduced in Section 2.1 of the main body of this paper. For the two excited-state equilibrium geometries, I also provide the magnitude of the mean hole-electron seperation vector[12] ($|\vec{d}_{h\to e}|$) of state S_1. Furthermore, Figure S3 shows the energy level diagram for fluorazene in the gas phase, based on the results of the DFT and TDDFT geometry optimizations.

As expected, there is only a single minimum on the PES of the singlet ground state; in Figures S2 and S3, it is labeled S_0-GS. At the ground-state equilibrium geometry, the heavy-atom skeleton of the molecule is near-planar, such that parameter $\mathrm{RMSD_{min}}$ takes a near-zero value. (The reason that the optimized geometry is not ideally planar, and the value of $\mathrm{RMSD_{min}}$ is not exactly zero, is because symmetry constraints were not imposed in the course of the optimizations. However, the deviation from planar geometry is very

slight.)

At this point, is informative to examine the vertical excitation spectrum of fluorazene that is predicted by the TD-CAM-B3LYP method. The lowest four singlet excited states are characterized in the upper part of Table S1. Accompanying this data, Figure S1 shows electron density difference maps (EDDMs) for these states. An EDDM is defined as the difference between the electron density of the given excited state, and that of the ground state, at the same nuclear geometry. Thus, the EDDM shows the redistribution of electron density associated with a vertical transition.

States S_1 and S_2 both show small hole-electron separation vectors; as such, these states are both locally excited in nature. Their locally excited character is also reflected by the structures of their EDDMs, which show little charge redistribution with respect to the ground state. State S_2 has by far the largest oscillator strength from among the four low-lying excited states, which means that it makes the main contribution to the first photoabsorption band of fluorazene.

States S_3 and S_4 are both ICT-type states with large hole-electron separation vectors. The inspection of their EDDMs confirms that these states involve a much larger amount of density redistribution than states S_1 and S_2. The EDDM for state S_3 indicates that this state corresponds to a shift of electron density from the C=C bonds of the pyrrole moiety onto atoms C6, C9, and, to a smaller extent, atoms C7 and C11. State S_4, on the other hand, exhibits charge transfer from the C=C bonds of the pyrrole moiety onto atoms C7, C8, C10, and C11. It shows a somewhat larger hole-electron separation than state S_3.

It is well documented that, in some aromatic and heteroaromatic molecules, TDDFT predicts an incorrect energy ordering of the low-lying $\pi\pi^*$-type excited states.[13–17] This problem can potentially come into play in the case of fluorazene. Therefore, in order to verify the spectrum predicted at the TD-CAM-B3LYP level of theory, I re-calculated it with the third-order algebraic-diagrammatic construction method[18,19] (ADC(3)) method. As with the TDDFT calculation, I performed the ADC(3) calculation with the program Q-Chem. For the sake of consistency with TD-CAM-B3LYP, I used the def2-SVP basis set with Cartesian d basis functions. I employed a restricted Hartree-Fock (RHF) reference determinant.

The vertical excitation spectrum obtained with ADC(3) is described in the lower part of Table S1. It can be seen that the state ordering is the same as with the TD-CAM-B3LYP method: state S_1 is the optically dark locally excited state, state S_2 is the optically bright locally excited state, while states S_3 and S_4 are both ICT-type states, with the state S_4 having a larger hole-electron separation than state S_3. The vertical excitation energies obtained at the TD-CAM-B3LYP level coincide, to within a few tenths of an electronvolt, with those calculated at the ADC(3) level. This suggests that TD-CAM-B3LYP provides a correct description of the relevant excited electronic states in the Franck-Condon region. I therefore move on to examine the performance of this methodology further away from the Franck-Condon geometry.

Table S1: Vertical excitation spectrum of fluorazene – excitation energies (ΔE) and associated oscillator strengths (f). $|\vec{d}_{h\rightarrow e}|$ is the mean hole-electron seperation vector.[12] Both the TD-CAM-B3LYP and the ADC(3) calculation were performed at the ground-state equilibrium geometry optimized at the CAM-B3LYP level. The labels LE-1, LE-2, ICT-1, and ICT-2 indicate the diabatic character of each excited state.

Method	Excited state	ΔE, eV	f	$\lvert\vec{d}_{h\rightarrow e}\rvert$, Å
TD-CAM-B3LYP	S_1 (LE-1)	5.134	0.0146	0.79
	S_2 (LE-2)	5.413	0.3150	0.83
	S_3 (ICT-1)	5.585	0.0650	2.51
	S_4 (ICT-2)	5.710	0.0592	3.26
ADC(3)	S_1 (LE-1)	4.717	0.0200	0.43
	S_2 (LE-2)	5.272	0.2972	0.60
	S_3 (ICT-1)	5.583	0.0441	2.12
	S_4 (ICT-2)	5.901	0.0487	3.15

Figure S1: EDDMs for the low-lying singlet states of fluorazene, calculated at the TD-CAM-B3LYP level. The EDDMs were visualized in the form of isosurfaces with isovalues of $\pm 0.005\ e/{a_0}^3$. The red and blue isosurfaces delimit regions in which the electron density is increased and decreased, respectively, relative to state S_0.

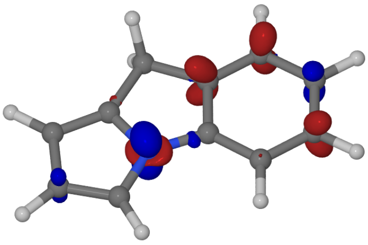

(a) S_1 (LE-1)

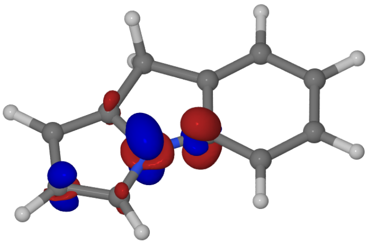

(b) S_2 (LE-2)

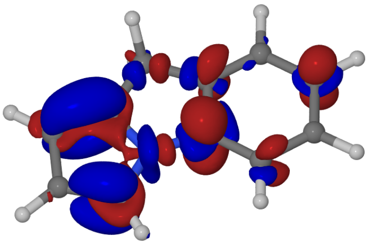

(c) S_3 (ICT-1)

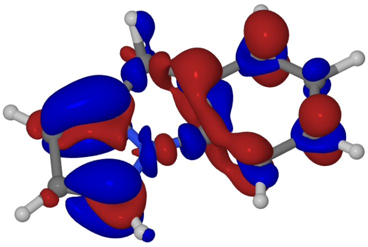

(d) S_4 (ICT-2)

I located two minima on the PES of state S_1. The first is the locally excited structure (S_1-LE), whose geometry is shown in Figure S2 (b). S_1-LE is characterized by a near-planar geometry, and by a small hole-electron seperation, which is the hallmark of a locally excited structure. At the S_1-LE minimum, the electronic state of the molecule is similar to the S_1 (LE-1) state at the Franck-Condon geometry (see Table S1).

The second minimum on the PES of state S_1 is the intramolecular charge transfer structure (S_1-ICT), which is illustrated in Figure S2 (b). A note on terminology: in the computational study by Galván and co-workers,[20] this excited-state minimum is referred to as the bent quinoidal (BQ) structure. (The reason this structure is said to be quinoidal is because bonds C7–C8 and C10–C11 are contracted relative to the ground-state geometry. For atom numbering, see Figure S10 in Section S3 later on in this document.) Accordingly, when referring specifically to the minimum-energy geometry, I use the designation S_1-ICT (BQ). The S_1-ICT (BQ) structure is distinguished by a partial pyramidalization of carbon atom C6, which causes a bending deformation of the heavy-atom skeleton. At the S_1-ICT (BQ) minimum, the electronic structure of state S_1 is analogous to the S_3 (ICT-1) state at the Franck-Condon geometry (see Table S1). Its ICT character is demonstrated by a large hole-electron separation.

The predictions of the TD-CAM-B3LYP level of theory can be compared to the results of previous theoretical studies of the photophysics of fluorazene and related compounds. The existence of the S_1-LE and S_1-ICT (BQ) minima on the PES of state S_1 of fluorazene is consistent with the results of previous simulation studies by Xu and co-workers[21] and by Galván and co-workers,[20] both of which used the complete active space self-consistent field[22] (CASSCF) method for geometry optimizations.

A point of note is that, according to my TD-CAM-B3LYP calculations for the isolated fluorazene molecule, there is only one ICT-type minimum on the PES of state S_1, and it is associated with a bent molecular geometry. Previously, Xu et al.[21] reported that the isolated molecule also has a second, planar, ICT-type structure (denoted the PICT structure), which lies slightly higher in energy than the bent ICT structure. Conversely, in a later study, Galván et al.[20] did not locate the PICT structure for the isolated molecule. However, these authors determined that the PICT structure (which they referred to as the linear quinoidal structure – LQ) does appear as a minimum when polar solvation is taken into account.[20] Thus, the fact that TDDFT does not predict the existence of a PICT-type minimum on state S_1 in the gas phase does not necessarily reflect negatively on the accuracy of that method, as it is unknown whether such a minimum exists under these conditions.

Further on the subject of the geometry of the ICT structure, He and Li[23] have previously reported an ICT-type minimum whose geometry is planar, and whose six-membered ring displays an anti-quinoidal deformation (bonds C7–C8 and C10–C11 are elongated with respect to the ground-state geometry). These authors relied on the CASSCF method for geometry optimizations, albeit with a smaller active space than that used in

Figure S2: Ground- and excited-state equilibrium geometries of fluorazene as optimized with the DFT and the TDDFT methods. The parameter $\mathrm{RMSD_{min}}$ is a quantitative measure of the deviation of the heavy-atom skeleton from a planar geometry. $|\vec{d}_{h\to e}|$ is the magnitude of the mean hole-electron seperation vector.[12] The lengths of bonds in the conjugated π-bonding system are marked in units of Ångström (Å).

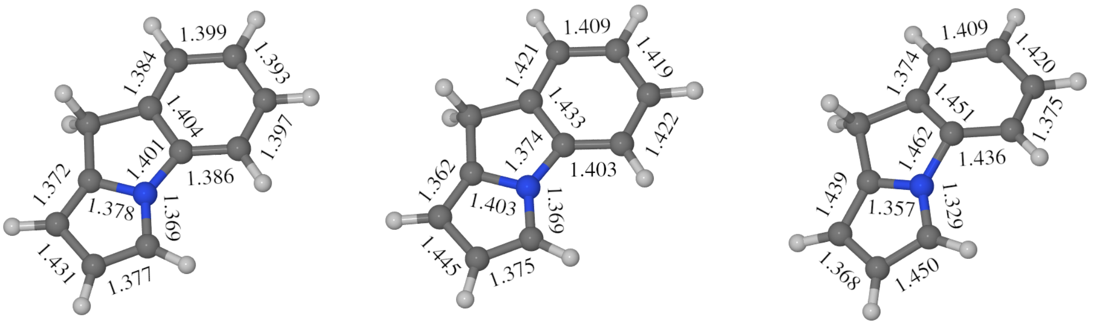

(a) S_0-GS
$\mathrm{RMSD_{min}} = 0.00$ Å

(b) S_1-LE
$\mathrm{RMSD_{min}} = 0.00$ Å
$|\vec{d}_{h\to e}| = 0.88$ Å

(c) S_1-ICT (BQ)
$\mathrm{RMSD_{min}} = 0.23$ Å
$|\vec{d}_{h\to e}| = 2.88$ Å

Refs. 20 and 21. The possibility of anti-quinoidal ICT structures in *N*-phenylpyrroles was also considered in theoretical studies by Zilberg and Haas[24] and by Cogan, Zilberg, and Haas.[25]

I have not located a comparable anti-quinoidal ICT-type minimum (whether planar or non-planar) for the isolated fluorazene molecule. Given that the existence of such a minimum is not supported by Refs. 20 and 21, I hypothesize that it represents an artifact of the reduced CASSCF active space employed by He and Li.[23] Still, my own calculations indicate that at least one anti-quinoidal ICT-type minimium exists on the PES of state S_1 of the fluorazene–3 ACN cluster (see Section S5 later on in this document). Evidently, some solvent configurations stabilize anti-quinoidal ICT structures. It follows that such structures may potentially be populated to some extent in the solution phase.

As discussed in more detail in the Background section of my paper, the dual fluorescence process of fluorazene involves a quasi-equilibrium between the LE structure, and one or more ICT-type structures. Now, according to the TDDFT calculations, the S_1-ICT (BQ) minimum lies 0.04 eV higher in energy than the S_1-LE minimum. (The energy difference includes zero-point vibrational energy – ZPVE – corrections.) Taken at face value, this result would imply that both these minima should be populated to a comparable extent already in a nonpolar environment. It would then follow that fluorescence emission from the S_1-ICT (BQ) minimum should be observed in a nonpolar solvent.

In reality, it is well documented that fluorazene exhibits dual fluorescence only in polar solvents, such as alkyl nitriles and tetrahydrofuran.[26,27] It is therefore clear that the TD-CAM-B3LYP level of theory artificially underestimates the energy difference between the S_1-ICT (BQ) minimum, and the S_1-LE minimum, though without the benefit of

Figure S3: Energy level diagram for the isolated fluorazene molecule as calculated at the CAM-B3LYP/def2-SVP and TD-CAM-B3LYP/def2-SVP level of theory. For the sake of clarity, only states S_0 to S_4 are included. The electronic state on whose PES the given structure was optimized is indicated with a bullseye symbol. The character of each excited state is indicated with the use of color. The arrows indicate vertical excitation into state S_2, which is the lowest bright excited state, and vertical fluorescence emission from the S_1-LE and the S_1-ICT (BQ) structures.

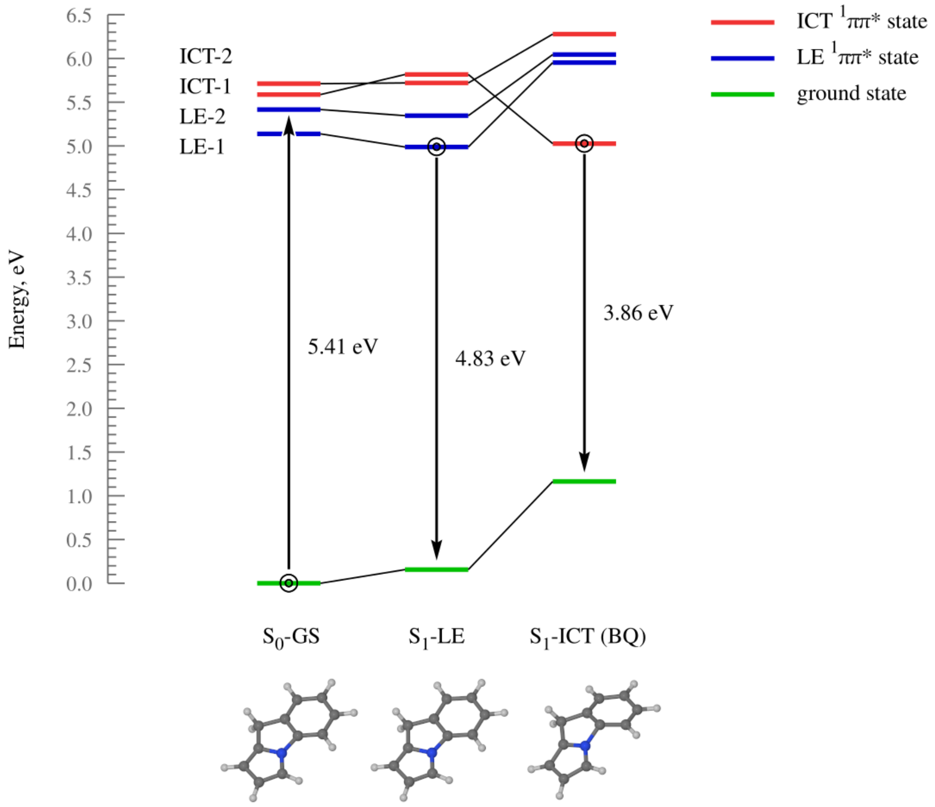

further, more accurate calculations, it is impossible to say by how much.

To gain a more quantitative insight into how TD-CAM-B3LYP describes interconversion between the S_1-LE minimum and S_1-ICT (BQ) minimum, I scanned the ground- and excited-state PESs along a reaction path which connects these two structures. The reaction path was generated through linear interpolation in internal coordinates (LIIC). Firstly, the initial and the final structures were described with use of a common internal coordinate system, which was set up automatically with the “define” subprogram of TURBOMOLE. Afterwards, the reaction path connecting the two structures was generated by linear interpolation in terms of this coordinate system. The resulting reaction path is illustrated in Figure S4. It can be seen that the LIIC procedure leads to a physically reasonable reaction path. In order to ensure that my results can be reproduced, the molecular geometries along the reaction path are included as part of the electronic Supporting Information.

The PES scan itself consisted of single-point calculations at molecular geometries along the interpolated reaction path. In order to have a benchmark for the TDDFT calculations,

Figure S4: Interpolated reaction path leading from the S_1-LE minimum to the S_1-ICT (BQ) minimum. The reaction path consists of 17 molecular geometries numbered 0 to 16, where the 0-th geometry is the S_1-LE structure, and the 16-th geometry is the S_1-ICT (BQ) structure. For the sake of clarity, only every fourth geometry is shown in this diagram, and successive geometries are drawn in different colors.

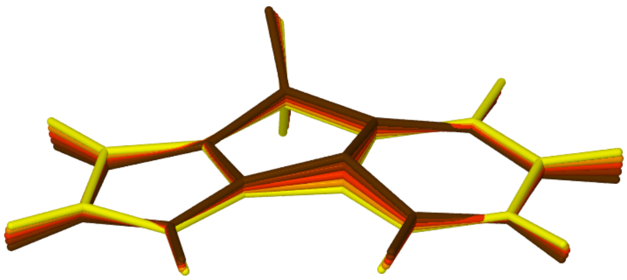

I subsequently re-calculated the single point energies with the ADC(3) method. The simulation parameters were the same as in the calculation of the vertical excitation spectrum.

The results of the PES scan are visualized in Figure S5. For the sake of clarity, the potential energy curves calculated with the TDDFT method are plotted in bright colors, while those obtained with the ADC(3) method are all plotted in black.

I discuss first the ADC(3) potential energy curves (Figure S5 (a)). As the molecule is displaced away from the S_1-LE minimum (the leftmost geometry in Figure S5 (a)), the energy of state S_1 rises, while the energies of states S_3 and S_4 decrease. As the molecule moves farther along the reaction path, state S_3 shows an avoided crossing with state S_2, and subsequently state S_2 undergoes an avoided crossing with state S_1. Near the S_1-ICT (BQ) minimum (the rightmost point along the reaction coordinate), state S_1 has acquired ICT-1 character. According to the ADC(3) method, the S_1-ICT (BQ) minimum lies 0.45 eV higher in energy than the S_1-LE minimum. (Here, I am referring to the minima optimized at the TD-CAM-B3LYP level of theory. ZPVE corrections are likewise taken from the TDDFT calculation.) This confirms my earlier conclusion that the TDDFT method underestimates the energy difference between the two structures.

The potential energy curves predicted by the CAM-B3LYP and TD-CAM-B3LYP methods are plotted in Figure S5 (b). In order to facilitate a comparison with the benchmark provided by the ADC(3) method, Figure S5 (c) shows the excited-state energies alone in a narrower energy range. The excited-state potential energy curves obtained with TDDFT are qualitatively similar to their ADC(3) counterparts, but the energy difference between the S_1-ICT (BQ) and the S_1-LE structures is smaller than at the ADC(3) level. As a consequence, at the TD-CAM-B3LYP level, the avoided crossing between states S_1 and S_2 appears earlier along the reaction coordinate than it does according to the

Figure S5: Energies of the lowest few electronic states of fluorazene along the interpolated reaction path leading from the S_1-LE minimum to the S_1-ICT (BQ) minimum, calculated with the (a) ADC(3) and the (b) TD-CAM-B3LYP methods. Panel (c) compares the potential energy curves of the singlet excited states obtained with the two methods. The origin of the energy axis corresponds to the energy of state S_0 at the ground-state minimum as optimized at the CAM-B3LYP level. (Within the ADC(3) method, the ground state is described at the third-order Møller–Plesset perturbation theory (MP3) level.)

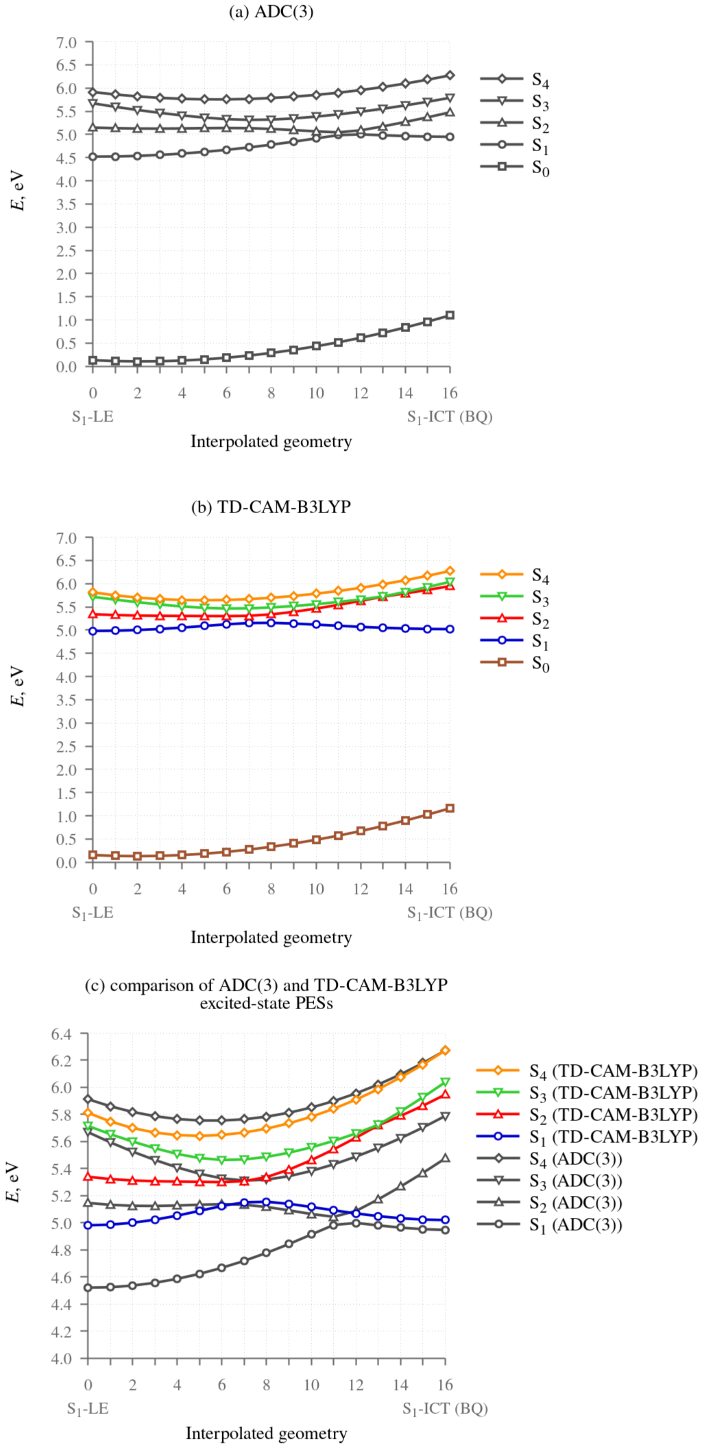

ADC(3) calculations.

In summary, the level of accuracy achieved by the TD-CAM-B3LYP method is best described as semi-quantitative: this method gives the correct energy ordering of the low-lying excited states at the Franck-Condon geometry, and the topography of the excited-state PESs is qualitatively correct. However, according to the benchmark provided by the ADC(3) method, the S_1-ICT (BQ) structure is artificially stabilized relative to the S_1-LE structure by ca. 0.4 eV. I consider this acceptable for my purposes, because the consequences of the error introduced by the TD-CAM-B3LYP level of theory are easy to predict: the quasi-equilibrium between the S_1-LE and the S_1-ICT (BQ) structures will be shifted in favor of the latter.

S1.2 Excited-State Absorption Transitions

In the second part of the benchmark calculations, I evaluated the performance of the TD-CAM-B3LYP level of theory in the calculation of excited-state absorption (ESA) transitions of fluorazene. Part of the reason this was necessary was that some of the relevant ESA transitions may lead to states with a significant contribution from doubly excited configurations,[28] which cannot be correctly described with the TDDFT method.

As regards the choice of benchmark method for the calculation of ESA transitions, my original intention was to use to the ADC(3) method also for this purpose. However, I found that attempting to calculate a large number of excited states invariably led to a convergence failure. For this reason, I resorted instead to using extended multi-state complete active space second-order perturbation theory[29,30] (XMS-CASPT2) as the benchmark for the TDDFT method.

The XMS-CASPT2 calculations were carried out as single-point calculations at excited-state geometries optimized at the TD-CAM-B3LYP level. I used the XMS-CASPT2 implementation in the program BAGEL,[31,32] version 1.1.2. The active space of the reference CASSCF calculation consisted of the 11 π- and π^*-type orbitals which arise mainly from carbon and nitrogen 2p atomic orbitals. The orbitals included in the active space are plotted in Figure S6. A state-averaging scheme was imposed in the reference CASSCF calculations with the inclusion of the lowest twelve singlet states (which is to say, states S_0 to S_{11}) with equal weights. A vertical shift of 0.5 E_h (hartree) was imposed.[33] Moreover, the so-called single-state single-reference (SS-SR) contraction scheme was used. The cc-pVDZ basis set[34] was employed in combination with the cc-pVDZ-jkfit density fitting basis set.[35,36]

When calculating the ESA transitions with the TDDFT method, I included excited states up to and including S_{20}. The reason for including more excited states in the TDDFT calculation than in the XMS-CASPT2 calculation has to do with the technical setup of the latter. Because the active space of the underlying CASSCF calculation only includes π- and π^*-type orbitals, the XMS-CASPT2 calculation only detects the ground state and $\pi\pi^*$-type excited states. The TDDFT calculation does not suffer from this limitation, and detects

Figure S6: CASSCF active space natural orbitals of fluorazene, depicted in the form of isosurfaces with isovalues of $\pm 0.05\ {a_0}^{-3/2}$. The orbitals were plotted at the S_1-LE minimum optimized at the TDDFT level. The choice of active space for the S_1-ICT (BQ) minimum is analogous.

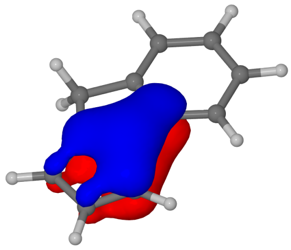

(a) MO36

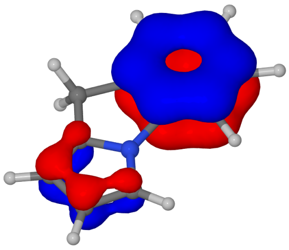

(b) MO37

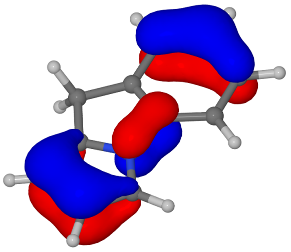

(c) MO38

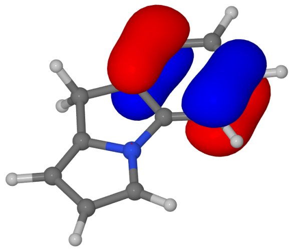

(d) MO39

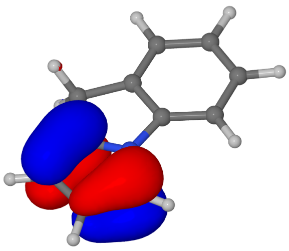

(e) MO40

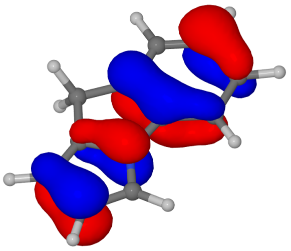

(f) MO41

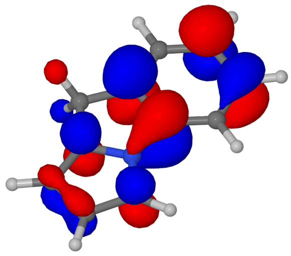

(g) MO42

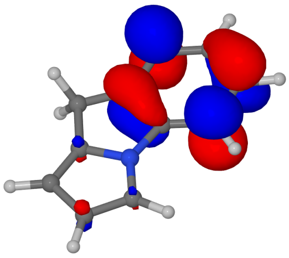

(h) MO43

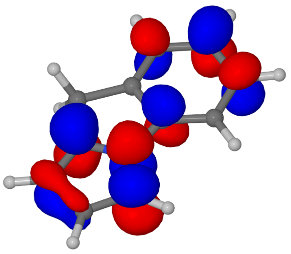

(i) MO44

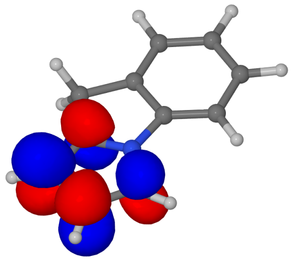

(j) MO45

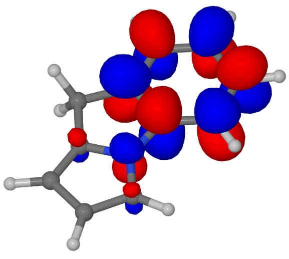

(k) MO46

$\pi\pi^*$-type states as well as states of other types ($\pi\sigma^*$-type, for instance). In order to achieve a balanced description between the two calculation, it is therefore necessary to include a larger number of states in the TD-CAM-B3LYP calculations.

The results of the calculation of the ESA transitions are summarized in Figure S7. I focus first on the ESA transitions at the S_1-LE minimum, which are shown in panel (a). Note that, in the difference spectrum, signal intensity is proportional to the squared magnitude of the transition dipole moment between the initial and the final states (see equation 3 in the main body of my paper). For this reason, I also use the squared magnitude of the transition dipole moment as a measure by which to compare the transition intensities predicted by different methods.

The XMS-CASPT2 calculation predicts that the S_1-LE structure shows two intense, narrowly spaced ESA transitions at energies of around 2.0 eV and 2.1 eV – these are the $S_1 \rightarrow S_5$ and the $S_1 \rightarrow S_6$ transitions. These two transitions can be identified with the sharp, intense ESA band that is seen in the TA spectrum of fluorazene at the probe pulse energy range of around 1.5–1.8 eV[27] (which corresponds to a wavelength range of roughly 850–700 nm).

The results of the TD-CAM-B3LYP calculation are partially in line with the XMS-CASPT2 benchmark – the TD-CAM-B3LYP calculation predicts as many as three strong transitions at around 2 eV, with the $S_1 \rightarrow S_6$ being very intense. At energies of around 3 eV and higher, the ESA transitions become very closely spaced in energy. This is because these transitions bring the molecule close to, or above, the photoionization threshold. The TDDFT calculation consequently detects a number of Rydberg-like states in which an electron is excited into semi-diffuse virtual orbitals. Transitions $S_1 \rightarrow S_{13}$ and $S_1 \rightarrow S_{18}$ are fairly intense, and they do not match up with any of the transitions detected by XMS-CASPT2. The remainder of the high-energy transitions have negligible intensity.

As a side note, the XMS-CASPT2 calculation is not visibly affected by the fact that some of the higher states are close to, or even above, the photoionization threshold. This is the active space only includes compact π- and π^*-type orbitals. Diffuse, Rydberg-like states are not detected.

Let us now move on to the S_1-ICT (BQ) minimum (Figure S7 (b)). The XMS-CASPT2 calculation predicts a moderately intense $S_1 \rightarrow S_8$ transition at around 2.8 eV, and a much stronger transition $S_1 \rightarrow S_9$ at around 3.5 eV. Experimentally, the ICT structure is fluorazene is known to show ESA signals at probe pulse energies of around 2.5 eV and 3.3 eV[27] (which corresponds to wavelengths of 495 nm and 370 nm). Thus, the calculation provides a reasonably good match for the observed ESA transition energies.

The TD-CAM-B3LYP calculation predicts only a single ESA transition with appreciable intensity, and that is the $S_1 \rightarrow S_7$ transition at roughly 2.8 eV. On the other hand, no intense transitions are found near 3.5 eV. As with the S_1-LE structure, for the S_1-ICT (BQ) structure the excited states at $S_1 \rightarrow S_n$ transition energies above around 3.0 eV become very narrowly spaced.

In conclusion, the TD-CAM-B3LYP level of theory is only partially successful at predicting the ESA transitions of fluorazene. However, it can still be used to model the TA spectrum, as long as that one focuses on the low-energy range of the spectrum (up to

around 3.0 eV), where the TDDFT calculation still performs reasonably well, and that one bears in mind the errors introduced by that method. More specifically, the TD-CAM-B3LYP method overestimates the intensities of at least some ESA transitions in the range of roughly 2–3 eV. The TDDFT calculation does not appear to be reliable for ESA transitions above around 3.0 eV, so the high-energy region of the TA spectrum must unfortunately be excluded from analysis.

Figure S7: Comparison of ESA transitions of fluorazene calculated with the XMS-CASPT2 and the TDDFT methods. The squared magnitude of the transition dipole moment of each transition is depicted as a vertical line.

(a) S_1-LE

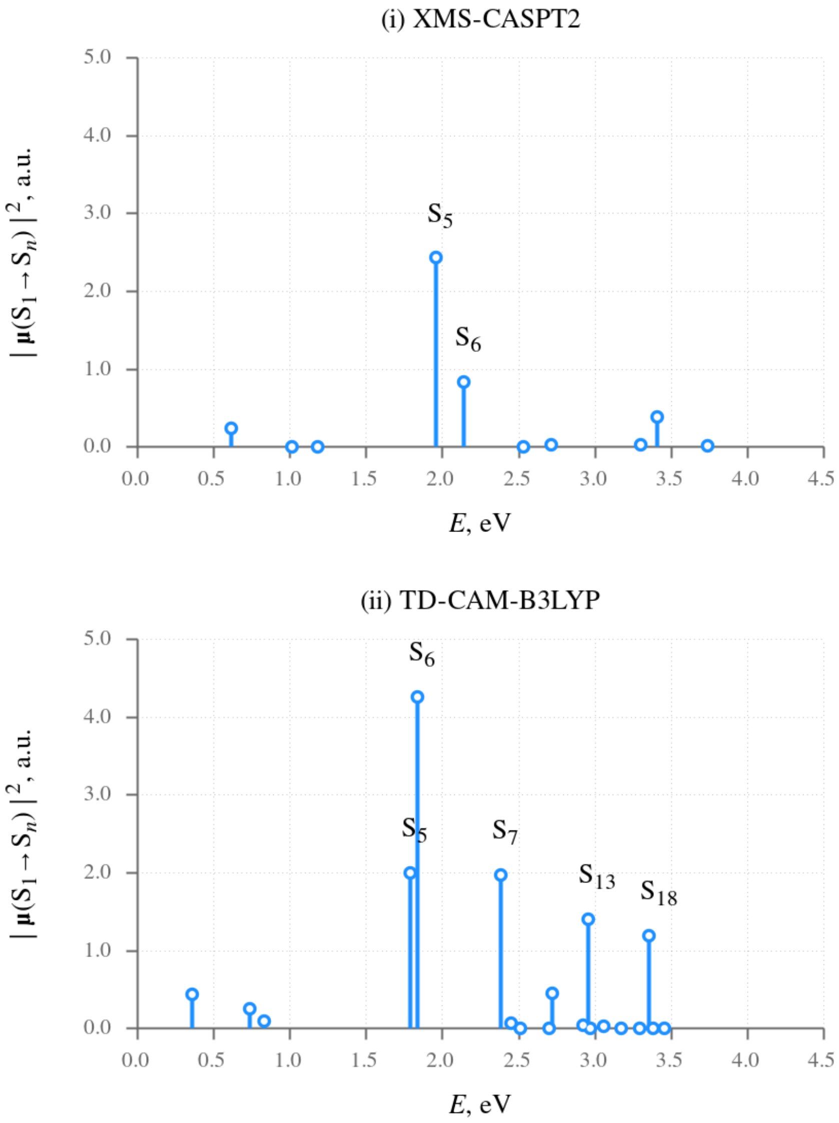

Figure S7 continued.

(b) S_1-ICT (BQ)

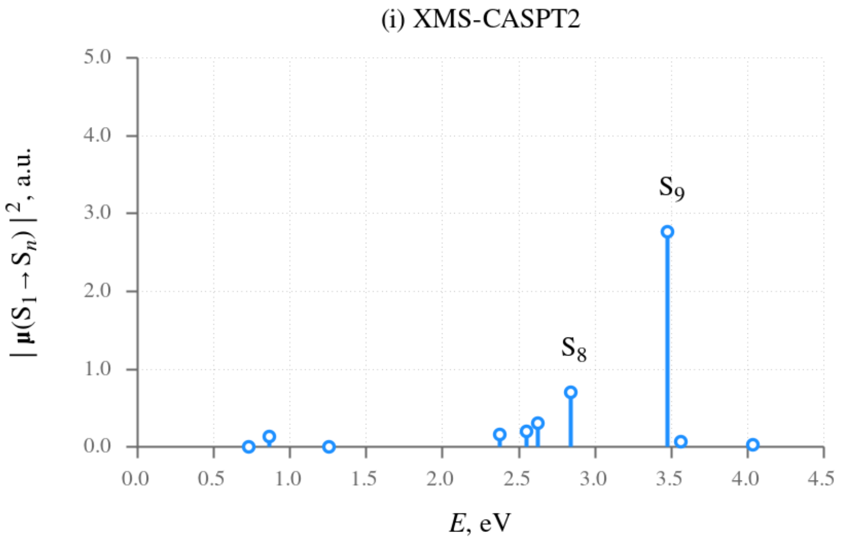

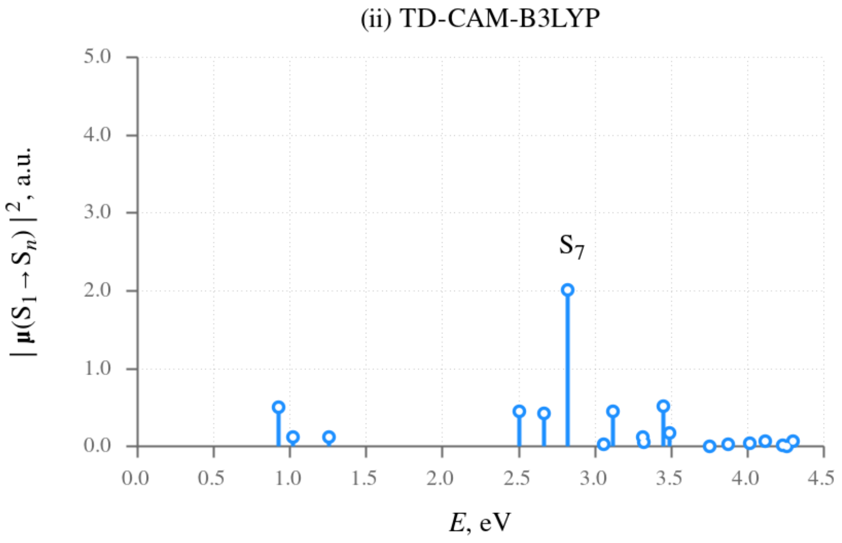

S2 Setup of NAMD Simulations

As stated in Section 2.1 of the main body of my paper, during the NAMD simulations the dilute solution phase was modeled by immersing a single fluorazene molecule in a nanodroplet of acetonitrile (ACN). The potential energy surfaces (PESs) of this system were constructed with the use of the hybrid quantum mechanics/molecular mechanics (QM/MM) method, and its dynamics was propagated with the fewest switches surface hopping algorithm. In the present section, I provide a more detailed description of this simulation setup.

S2.1 QM/MM Calculations

In the QM/MM method,[37–41] the system under study (denoted $\mathbb{S}$) is partitioned into two subsystems which are treated at different levels of approximation. The electronic structure of the inner subsystem ($\mathbb{I}$) is explicitly included in the simulation, and it is calculated on-the-fly with the use of a quantum-mechanical (QM) method. The outer subsystem ($\mathbb{O}$), in turn, is described with a molecular mechanics (MM) force field. When studying the photophysics of compounds in the dilute solution phase, the natural choice of QM/MM partitioning is to include the solute molecule is included in the inner subsystem, and to have the surrounding solvent molecules comprise the outer subsystem. This situation is shown in Figure 2 in the main body of my paper.

Because periodic boundary conditions are not available in TURBOMOLE or in Q-Chem, in my simulations the solution phase was represented by placing the solute molecule at the center of a spherical ACN nanodroplet containing 500 solvent molecules. Experimental data suggests that a 500-molecule solvent droplet is more than large enough for the purpose of modeling the dual fluorescence of *N*-phenylpyrroles. Namely, Schweke and Haas[42] have measured the fluorescence emission spectra of *N*-phenylpyrrole (NPP) in cryogenic argon matrices doped with ACN. These authors reported that already a single adjacent ACN molecule is sufficient to bring out the anomalous fluorescence of NPP.[42]

The polarization of the fluorophore by a polar solvent is crucial to the phenomenon of dual fluorescence. In order to be able to account for this effect, I employed the additive variant of the QM/MM method with electrostatic embedding. In this approach, the potential energy function ($E_{\text{QM/MM}}(\mathbb{S})$) of the system is broken down as follows:

$$E_{\text{QM/MM}}(\mathbb{S}) = E_{\text{QM}}(\mathbb{I}) + E_{\text{MM}}(\mathbb{O}) + E_{\text{QM-MM}}(\mathbb{I}, \mathbb{O}) \tag{1}$$

Here, $E_{\text{QM}}(\mathbb{I})$ represents the energy of the inner subsystem calculated at the QM level of theory. $E_{\text{MM}}(\mathbb{O})$ is the energy of the outer subsystem, evaluated at the MM level of theory. Lastly, $E_{\text{QM-MM}}(\mathbb{I}, \mathbb{O})$ is the QM-MM coupling term, whose purpose is to describe the interactions between the inner and outer subsystems.

In the present case, the inner and outer subsystems only interact via electrostatic and Van der Waals interactions. (There are no covalent bonds across the QM/MM boundary.) The electrostatic interactions are handled with the so-called electrostatic embedding scheme.[37]

That is to say, the point charges of the MM force field are included in the QM Hamiltonian. This measure allows the electronic structure of the solute to adapt to the charge distribution of its solvent. (Note that, in my simulations, polarization only works one way – the solvent is described using the non-polarizable OPLS-AA[43] force field.)

Because the electrostatic part of the interaction between the inner and outer subsystems is calculated during the QM calculation, it is subsumed into the term E_{QM}, which consequently becomes a function of $\mathbb{O}$ in addition to being a function of $\mathbb{I}$:

$$E_{\text{QM/MM}}(\mathbb{S}) = E_{\text{QM}}(\mathbb{I}, \mathbb{O}) + E_{\text{MM}}(\mathbb{O}) + E_{\text{QM-MM}}(\mathbb{I}, \mathbb{O}) \tag{2}$$

The electronic structure of fluorazene (the term $E_{\text{QM}}(\mathbb{I}, \mathbb{O})$) was calculated with the TDDFT method. The relevant simulation settings are given in the main body of this paper.

As for the solvent, the term $E_{\text{MM}}(\mathbb{O})$ was evaluated with the all-atom optimized potentials for liquid simulations[43] (OPLS-AA) force field. The parameters for ACN were sourced from Ref. 44.

The Van der Waals interactions between the solute and the solvent are formally included in the term $E_{\text{QM-MM}}(\mathbb{I}, \mathbb{O})$. For the sake of consistency with the OPLS-AA force field, these interactions were modeled with the 6–12 Lennard-Jones potential:

$$E_{\text{QM-MM}}(\mathbb{I}, \mathbb{O}) = \sum_{i \in \mathbb{I}} \sum_{j \in \mathbb{O}} 4\varepsilon_{ij} \left[\left(\frac{\sigma_{ij}}{r_{ij}} \right)^{12} - \left(\frac{\sigma_{ij}}{r_{ij}} \right)^{6} \right] \tag{3}$$

The van der Waals parameters for fluorazene were likewise taken from the OPLS-AA force field. The assignment of atom types is indicated in Figure S8. The corresponding interaction parameters are listed in Table S2. As per the usual convention, the "mixed" interaction parameters σ_{ij} and ε_{ij} which appear in equation 3 were obtained with the use of geometric combining rules: $\sigma_{ij} = (\sigma_{ii}\sigma_{jj})^{1/2}$ and $\varepsilon_{ij} = (\varepsilon_{ii}\varepsilon_{jj})^{1/2}$.

Figure S8: The assignment of OPLS-AA atom types to atoms comprising the fluorazene molecule.

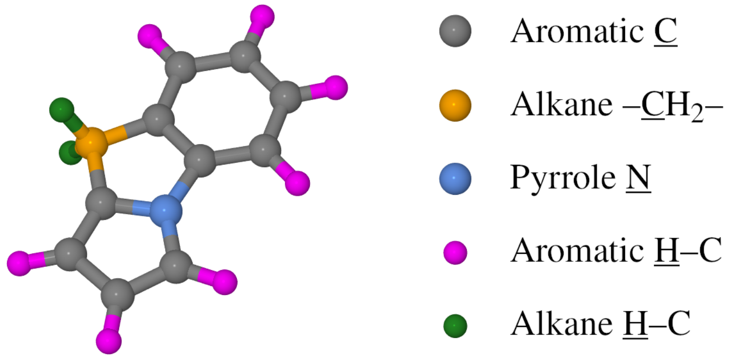

Table S2: 6–12 Lennard-Jones interaction parameters for fluorazene, adapted from the OPLS-AA force field.[43,44]

Atom type (i)	ε_{ii}, kcal/mol	σ_{ii}, Å
Aromatic C̲	0.0700	3.5500
Alkane –C̲H$_2$–	0.0660	3.5000
Pyrrole N̲	0.1700	3.2500
Aromatic H̲–C	0.0300	2.4200
Alkane H̲–C	0.0300	2.5000

In order to ensure that the solvent droplet remained roughly spherical in shape, and also to prevent the evaporation of the solvent, all atoms contained in the system were subjected to a harmonic spherical boundary potential:

$$V(\mathbf{R}) = \begin{cases} \frac{1}{2} k_\mathrm{B} \left(|\mathbf{R}| - R_\mathrm{B} \right)^2 & \text{if} \quad |\mathbf{R}| - R_\mathrm{B} > 0 \\ 0 & \text{otherwise} \end{cases} \tag{4}$$

The radius R_B was set to 21.85 Å, so as to maintain the density of the droplet near 786 kg/m^3 (the density of bulk ACN at room temperature and atmospheric pressure). The value of the force constant k_B was set to $1 \times 10^{-4}\ E_\mathrm{h}/a_0{}^2$.

The MM part of the QM/MM calculation, and the calculation of the QM/MM energy and gradients via equation 2, was performed by the "wrapper" program which performs the overall NAMD simulation. For details of the implementation, see Section S2.3 later on.

S2.2 Initial Conditions for NAMD Simulations

The initial conditions for the NAMD simulations were defined in such a way as to reproduce, insofar as possible, the experimental conditions used by Druzhinin and co-workers.[27] Setting up the simulations was a fairly complex, multi-stage procedure. This was in part because the system under study is described using the hybrid QM/MM method, such that the solute and the solvent are treated on a different footing. I followed the de facto standard practice in NAMD simulations of photoinduced processes in the solution phase, in which ZPVE energy is imparted on the solute molecule, but not on the solvent.[45,46]

The first step was to generate the nuclear positions and velocities of the fluorazene molecule. To this end, the ground-state minimum-energy geometry of fluorazene was optimized at the CAM-B3LYP level, and its normal modes were calculated numerically. Afterwards, 1000 phase space points (which is to say, 1000 sets of nuclear positions and velocities) were sampled from the Wigner distribution. As pointed out in Refs. 47– 49, semiclassical simulations are prone to an artificial leakage of zero-point vibrational energy from the stretching modes of hydrogen-heavy atom bonds to other vibrational modes. In

order to alleviate this problem, when generating the Wigner distribution, I froze the nine vibrational modes with the highest frequencies, which correspond to C–H stretching modes.

At the second stage of the procedure, the solvent was equilibrated with the solute. This was achieved by placing the fluorazene molecule (the ground-state minimum-energy geometry) at the center of a 500-molecule ACN nanodroplet. Afterwards, the solvent was equilibrated with the solute by propagating a molecular dynamics trajectory in the canonical (NVT) ensemble.

During the equilibration, the geometry of the fluorazene molecule was kept frozen, and the potential energy surface of the entire system was calculated at the MM level of theory. The charge distribution of the solute was represented by a set of point charges placed at the nuclear positions. These charges were obtained by fitting them to the electrostatic potential generated by the fluorazene molecule. The fit was performed with the Merz-Singh-Kollman (MKS) scheme[50] implemented in Gaussian 16.[3] The temperature of the solvent was maintained at 298 K by imposing the Langevin thermostat with a friction coefficient of $\gamma = 1\ \text{ps}^{-1}$. The Langevin equation of motion was propagated with the use of the Brünger-Brooks-Karplus (BBK) integrator[51] with a time step of 0.5 fs. The equilibration period lasted for 100 ps.

Following the equilibration period, 1000 solvent configurations were sampled from the molecular dynamics simulation at intervals of 2 ps, and were combined with the solute geometries generated previously by sampling from the Wigner distribution. In effect, this means that the solvent was equilibrated with the minimum-energy geometry of fluorazene, and not with the individual geometries sampled from the Wigner distribution, which include vibrational displacements from the minimum-energy geometry. This approximation is acceptable for a relatively rigid molecule such as fluorazene.

The 1000 combined solvent-solute geometries were used as the basis for the simulation of the photoabsorption spectrum of fluorazene with the nuclear ensemble method.[52,53] In the calculation of the spectrum, a Gaussian line shape function was used with a variance of $\sigma^2 = 0.04\ \text{eV}^2$. In Figure S9, the resulting spectrum is compared to the experimental spectrum of fluorazene in ACN solution.[26] It can be seen that, in the simulated spectrum, the first photoabsorption band is blue-shifted by roughly 0.8 eV with respect to the observed spectrum. This is in part because the calculation was performed with the relatively small def2-SVP basis set.

The fact that the calculated spectrum is blue-shifted with respect to experiment has implications for the choice of initial conditions. The TA experiments reported in Ref. 27 were performed with a pump pulse wavelength of 290 nm, which corresponds to a photon energy of 4.28 eV. (Some experiments were also performed with photoexcitation at 266 nm, but their results were reported to be similar to those with the longer wavelength.[27]) Because the TDDFT method overestimates the energy range of the first photoabsorption band of fluorazene, I must also assume a higher photon energy of the pump pulse. Accordingly, I decided to set the pump pulse photon energy to 5.1 ± 0.1 eV.

Phase space points (sets of nuclear positions and velocities along with an initial adiabatic state from S_1 to S_4) with excitation energies in the range 5.1 ± 0.1 eV were sampled with probabilities proportional to their oscillator strengths. Note that this procedure does not not explicitly model the interaction of the system with the pump pulse. Rather, the initial conditions are chosen in such a way as to represent the system having been photoexcited with the pump pulse. A total of 60 phase space points were selected in this way, and each was used as the starting point for a single NAMD trajectory. The simulations were propagated for a time period of 1.5 ps.

Figure S9: Simulated and observed[26] photoabsorption spectrum of fluorazene in ACN. The shaded area below the calculated photoabsorption curve represents the energy interval from which initial conditions for the nonadiabatic molecular dynamics simulations were sampled.

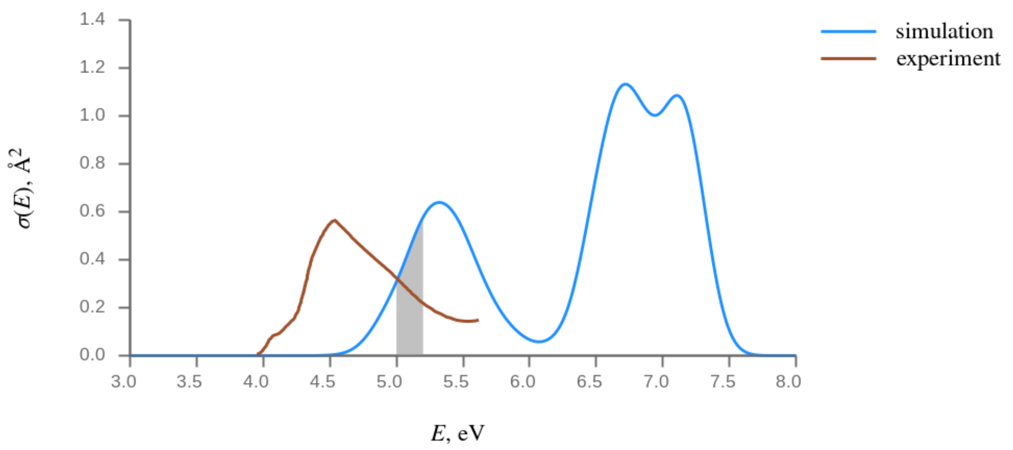

S2.3 NAMD Algorithm

This section covers the simulation settings which pertain to the dynamics of the system under study. The time-evolution of the system was propagated with the well-known fewest switches surface hopping[54–56] (FSSH) algorithm. In this approach, the nuclear wavepacket of the system is represented by a set of mutually independent semiclassical trajectories. In each simulated trajectory, the nuclei are described by means of classical mechanics, while the electronic structure of the molecule is treated quantum mechanically.

The wavefunction $\Psi(\mathbf{r}, t; \mathbf{R})$ of the electrons along a given nuclear trajectory $\mathbf{R} = \mathbf{R}(t)$ is expressed in terms of a linear combination of adiabatic states $\{\psi_j(\mathbf{r}; \mathbf{R})\}$ with time-dependent complex coefficients $\{a_j(t)\}$:

$$\Psi(\mathbf{r}, t; \mathbf{R}) = \sum_j a_j(t)\, \psi_j(\mathbf{r}; \mathbf{R}) \tag{5}$$

$|a_j(t)|^2$, the square modulus of the coefficient of the j-th state, is interpreted as the population of that state in the given trajectory.

The requirement that $\Psi(\mathbf{r}, t; \mathbf{R})$ satisfies the time-dependent electronic Schrödinger equation leads to a system of coupled differential equations for the time-evolution of the expansion coefficients:

$$\mathrm{i}\hbar\dot{a}_k = \sum_j a_j \left(\delta_{kj} E_k(\mathbf{R}) - \mathrm{i}\hbar\dot{\mathbf{R}} \cdot \mathbf{d}_{kj}\right) \tag{6}$$

where δ_{jk} denotes the Kronecker delta, $E_k(\mathbf{R})$ is the PES of the k-th adiabatic state, and $\mathbf{d}_{kj}$ is the nonadiabatic coupling vector (NACV) between states k and j:

$$\mathbf{d}_{kj} = \langle \psi_k(\mathbf{r}; \mathbf{R}) | \nabla_{\mathbf{R}} | \psi_j(\mathbf{r}; \mathbf{R}) \rangle \tag{7}$$

In each simulated trajectory, at any given time, one adiabatic state from among those included in the linear expansion 5 is singled out as the occupied state (or, the current state). The nuclei move according to the classical equations of motion on the PES of that state:

$$\ddot{\mathbf{R}}_A = -\frac{1}{M_A} \nabla_A E_n(\mathbf{R}) \tag{8}$$

Nonadiabatic effects are accounted for by allowing a trajectory to undergo a switch (or "hop") between the current state and another adiabatic state, which then becomes the new current state for the given trajectory. The switches are imposed stochastically on the basis of changes in the state populations.

In the present case, the linear expansion 5 included states from S_1 to S_4. The system of equations 6 was integrated with the use of the fourth-order Runge-Kutta method with a time step of 0.0004 fs, using quantities interpolated linearly between successive classical steps. Moreover, the time-evolution of the expansion coefficients was corrected for decoherence via the scheme proposed by Granucci and Persico.[57] The correction constant was set to $C = 0.1\ E_{\mathrm{h}}$ (hartree).

The dynamics of the nuclei (equation 8) was propagated with the use of the velocity Verlet integrator with a time step of 0.5 fs.

NACVs between excited states were calculated via the pseudo-wavefunction approach with the inclusion of electron translation factors.[58] On the subject of the NACVs, the phases of electronic wavefunctions calculated by a quantum chemistry program such as Q-Chem are set arbitrarily, and they can change from one time step to another. In order to correct for this effect, the wavefunction phases were monitored by calculating the normalized dot products between NACVs calculated in successive time steps. Whenever a phase change was detected, the NACV was multiplied by -1 from that point onward.

When the system underwent a hop from one state to another, the velocities of the QM atoms were rescaled uniformly in such a way as to conserve the total energy of the system. (In the case of upward hops, if the QM subsystem did not have sufficient kinetic energy to allow the hop, it was rejected.) The velocities of the MM atoms were always left unchanged. This measure prevents an unphysical transfer of energy between the inner and outer

subsystems during a nonadiabatic transition.

On the technical side, I carried out the NAMD simulations with a C++ program which acts as a "wrapper" around TURBOMOLE and Q-Chem. I originally developed this program to perform NAMD simulations with the spin-flip variant of TDDFT,[59,60] and I subsequently added an implementation of the QM/MM method.[61,62] At each time step of an NAMD trajectory, the wrapper generates input files for TURBOMOLE (for the calculation of state energies and the gradient of the occupied state) and for Q-Chem (for the calculation of NACVs). Afterwards, the wrapper runs TURBOMOLE and Q-Chem, parses the output files, and extracts the relevant quantities. A subroutine of the wrapper also calculates the molecular mechanics (MM) terms of the QM/MM energies and gradients. These quantities are then used to propagate the nuclear and electronic equations of motion.

The source code of the wrapper program is available for download from the Zenodo repository: https://doi.org/10.5281/zenodo.12759064.

S3 Origin of Ultrafast $S_2 \rightarrow S_1$ Internal Conversion

A consequence of equation 6 is that internal conversion (population transfer between electronic states of the same multiplicity) is driven by the motion of the nuclei parallel, or antiparallel, to the NACV between the initial and the final states. Thus, the calculation of the NACV provides a means of identifying the specific nuclear motions that are responsible for internal conversion. Presently, I will take advantage of this property in order to identify the specific vibrational modes that bring about the rapid $S_2 \rightarrow S_1$ internal conversion process of fluorazene.

Figure S10 shows the NACV between states S_2 and S_1 at the Franck-Condon geometry (which is to say, at the ground-state equilibrium geometry). It can be seen that the largest components of the NACV lie in the symmetry plane of the molecule, and they are situated on the carbon atoms comprising the phenyl ring, on the nitrogen atom, and on carbon atoms C2 and C5. It follows that the internal conversion process is mainly caused by deformation modes (bond stretching and bending) of the six-membered ring, and the stretching of bonds N1–C6, N1–C2, and N1–C5. This observation also explains the short timescale of the internal conversion process: the vibrational periods of C–C and N–C bonds are on the order of 20 fs. Note that neither the pyramidalization of atom C6, nor the out-of-plane bending of the heavy-atom skeleton as a whole, play a direct role in the internal conversion process.

Figure S10: NACV between states S_2 and S_1 of fluorazene at the Franck-Condon geometry. The NACV was calculated analytically with the TDDFT method. Atom numbering is shown in red.

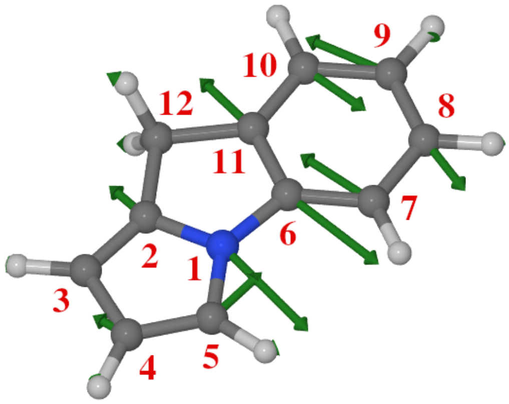

S4 Transition State for S_1-LE–S_1-ICT (BQ) Isomerization

The existence of two minima on the PES of state S_1 implies that there is also a transition state (a first-order saddle point) on the minimum-energy path connecting them. I have undertaken to optimize this transition state at the TD-CAM-B3LYP level of theory. On the technical side, the calculation was performed using the program Q-Chem interfaced to Gaussian 16 (see Section S1 above). This setup enabled us to take advantage of the Berny algorithm implemented in Gaussian 16. The initial guess for the geometry of the transition state was taken from the LIIC reaction path, which was discussed previously in Section S1.

The resulting transition state geometry is illustrated in Figure S11 (a). Moreover, Figure S11 (b) shows the nuclear displacements associated with the mode with the imaginary frequency. For the sake of brevity, I will refer to the transition state structure as S_1-TS.

As can be seen in Figure S11 (a), S_1-TS is characterized by a partially bent geometry. The mode with the imaginary frequency is dominated by the out-of-plane displacement of atom C6. Taking into account ZPVE corrections, S_1-TS lies only 0.04 eV higher in energy than the S_1-LE minimum. This finding is qualitatively consistent with the results of the PES scan reported previously in Section S1.1, which showed that the TD-CAM-B3LYP level of theory predicts an overly flat S_1 PES. In reality, both the S_1-ICT (BQ) minimum and the S_1-TS structure most likely lie a few tenths of an electronvolt higher in energy, relative to the S_1-LE minimum, than is predicted by the TDDFT calculation.

Figure S11: (a) Molecular geometry of the S_1-TS structure. Selected bond lengths are given in units of ångström (Å). (b) Atomic displacements corresponding to the single mode with an imaginary frequency.

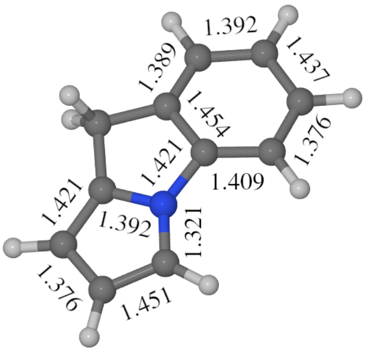

(a) geometry of S_1-TS

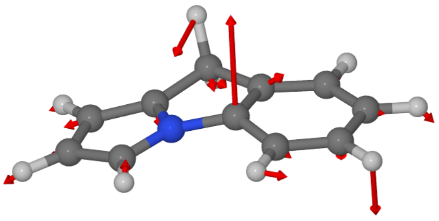

(b) normal mode with imaginary frequency

S5 Geometry of the Microsolvated ICT Structure

As noted in Section 3.1 in the main body of my paper, there is seemingly a discrepancy between the geometry optimizations for the isolated fluorazene molecule, and the results of the NAMD simulations. Namely, the geometry optimizations reported in Section S1.1 of this document indicate that, in the isolated molecule, the only ICT-type minimum on the PES of state S_1 is the markedly non-planar (bent) S_1-ICT (BQ) structure. One might therefore expect that the geometry of the ICT structure in the polar solution phase should likewise be bent. However, in the NAMD simulations of the relaxation dynamics of fluorazene in the ACN nanodroplet, near-planar ICT structures are more prevalent than non-planar (bent) ICT structures.

Aiming to understand why the two sets of simulations give different predictions as to the geometry of the ICT structure, I investigated how the geometry is affected by the presence of the solvent. For the sake of methodological simplicity, in these calculations I used the fluorazene−3 ACN cluster as a model of interactions in the bulk solution phase. Small molecular clusters of this type have often been used in theoretical studies of solvent-solute interactions in the excited state.[63–68] Moreover, I performed these calculations with the all-QM approach – which is to say, I explicitly treated the electronic structure of the solute and the solvent at the TD-CAM-B3LYP level of theory. Using only three ACN molecules represents a compromise between the realism of the model, and computational tractability.

The geometry optimizations for the fluorazene−3 ACN clusters were performed within the program Gaussian 16. In order to narrow down the scope of the calculations, I focused exclusively on ICT-type structures; I did not attempt to optimize either ground-state structures, or LE-type excited-state structures. The initial geometries for the geometry optimizations were constructed in such a way as to stabilize the ICT state: the ACN molecules were placed roughly parallel to the long axis of the fluorazene molecule. Their nitrile groups were oriented towards the pyrrole moiety of fluorazene, and their methyl groups were oriented towards the phenyl moiety.

The simulation parameters were analogous as in the isolated-molecule calculations, with the important exception that the PES of state S_1 was corrected for dispersion interactions via the D3 scheme of Grimme and co-workers with Becke-Johnson damping.[69] As per the default in Gaussian 16, the "UltraFine" integration grid was used. The optimized geometries were confirmed to correspond to minima on the PES of state S_1 through analytical calculations of vibrational frequencies.[70,71]

The electronic structures of the resulting ICT-type minima of the fluorazene−3 ACN cluster were characterized by plotting the EDDM of state S_1. Furthermore, for each minimum, I calculated values of $\mathrm{RMSD}_{\mathrm{min}}$ and of the mean hole-electron seperation vector. The latter calculation was done in Q-Chem at the minimum-energy geometries optimized with Gaussian 16.

I have located eight ICT-type minima on the PES of state S_1 of the fluorazene−3 ACN cluster. I label them **1** to **8** in order of increasing (ZPVE-corrected) energy. Their

geometries are shown in Figure S12.

In all of the clusters that I have located, the EDDM of state S_1 is almost completely localized on the fluorazene molecule. Thus, intermolecular charge transfer between the solute and the solvent does not occur to any significant extent.

In clusters **1**, **2**, and **4** through **8**, state S_1 involves ICT from the C=C bonds of the pyrrole moiety of fluorazene onto atoms C6, C9, and, to a smaller extent, C7 and C11. The electronic state of the molecule is therefore similar to the S_3 (ICT-1) state of the isolated fluorazene molecule at the Franck-Condon geometry (see Table S1 earlier on in this document). These seven clusters all show varying degrees of bending of the molecular skeleton, though in clusters **1**, and **6**, the fluorazene molecule is much closer to planar than in the isolated S_1-ICT (BQ) structure. (More specifically, the value of $\mathrm{RMSD}_{\mathrm{min}}$ is lower than for the S_1-ICT (BQ) structure of the isolated molecule – see Figure S1 earlier on in this document.) Tellingly, none of these seven clusters shows a stronger bending (or, a higher value of $\mathrm{RMSD}_{\mathrm{min}}$) than the S_1-ICT (BQ) structure of the isolated molecule. These observations suggest that some solvent configurations favor a partial planarization of the bent ICT state.

An interesting effect is seen in cluster **3**, where state S_1 has a different diabatic character than in the other clusters that I have found – it is very similar to the S_4-(ICT-2) state of the isolated molecule at the Franck-Condon geometry (see Table S1). In cluster **3**, the deformation of the phenyl ring is anti-quinoidal. In other words, the solvent configuration seen in cluster **3** induces a crossover from ICT-1-like character to ICT-2-like. (The crossover from one ICT-type state to the other is also confirmed by the magnitude of the mean hole-electron seperation vector, which is larger for cluster **3** than for any of the other clusters.) The ICT-2-like state apparently favors a planar geometry, and so the heavy-atom skeleton of the fluorazene molecule in cluster **3** is close to planar.

In summary, I have determined that the geometry of the ICT structure of fluorazene is sensitive to the presence, and the positioning, of solvent molecules. There are two qualitatively distinct effects at play. Firstly, some solvent configurations cause a partial planarization of heavy-atom skeleton. Secondly, one solvent configuration from among those which I have located induces a switch from one ICT state to another, and a near-complete planarization of the heavy-atom skeleton. These findings can be extrapolated to the NAMD simulations, in which the fluorazene molecule is interacting with a much larger number of ACN molecules. In the course of these simulations, the solvent shell is continually fluctuating, and the fluorazene molecule presumably experiences both of the above-mentioned effects at different times. This explains the prevalence of near-planar ICT geometries in the simulated trajectories.

The minimum-energy geometries of the eight fluorazene–3 ACN clusters are provided as part of the electronic Supporting Information.

Figure S12: Catalogue of ICT-type fluorazene−3 ACN clusters. E is the (ZPVE-corrected) energy of the given cluster relative to the lowest-energy cluster from among those that I have located (cluster **1**). On the right-hand side, the EDDM of state S_1 of each cluster was visualized in the form of isosurfaces with isovalues of $\pm 0.005\ e/a_0{}^3$. The red and blue isosurfaces delimit regions in which the electron density is increased and decreased, respectively, in state S_1 relative to state S_0.

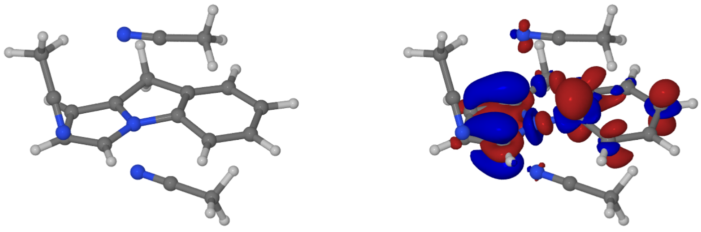

(a) cluster **1**:
$E = 0$ eV, $\mathrm{RMSD_{min}} = 0.12$ Å, $|\vec{d}_{h\to e}| = 3.03$ Å

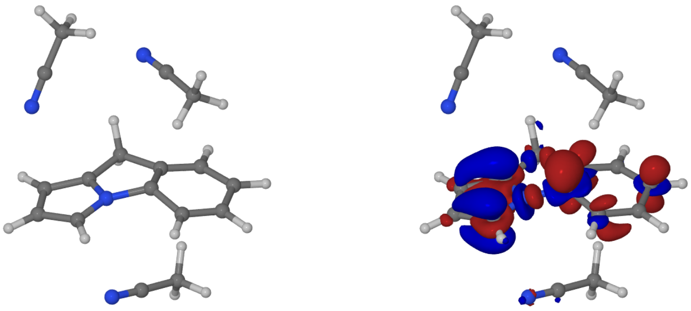

(b) cluster **2**:
$E = 0.012$ eV, $\mathrm{RMSD_{min}} = 0.18$ Å, $|\vec{d}_{h\to e}| = 3.05$ Å

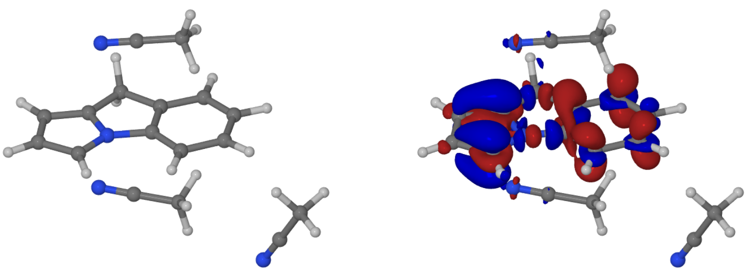

(c) cluster **3**:
$E = 0.034$ eV, $\mathrm{RMSD_{min}} = 0.02$ Å, $|\vec{d}_{h\to e}| = 3.53$ Å

Figure S12 continued.

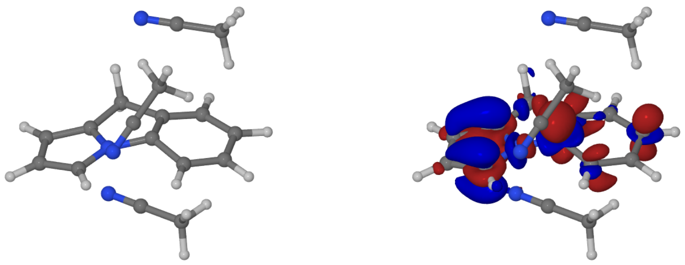

(d) cluster **4**:
$E = 0.035$ eV, $\mathrm{RMSD_{min}} = 0.17$ Å, $|\vec{d}_{h\to e}| = 3.08$ Å

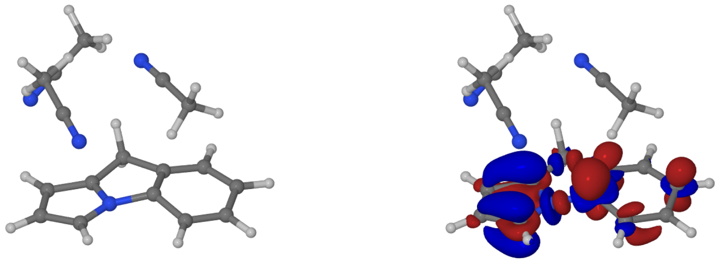

(e) cluster **5**:
$E = 0.036$ eV, $\mathrm{RMSD_{min}} = 0.14$ Å, $|\vec{d}_{h\to e}| = 3.02$ Å

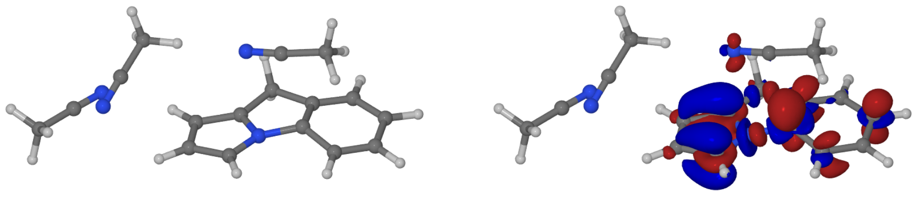

(f) cluster **6**:
$E = 0.120$ eV, $\mathrm{RMSD_{min}} = 0.13$ Å, $|\vec{d}_{h\to e}| = 2.94$ Å

Figure S12 continued.

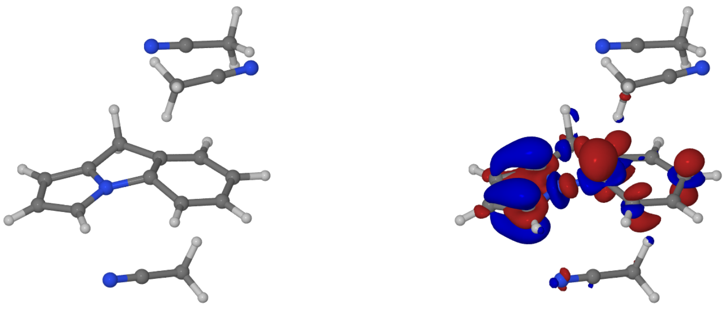

(h) cluster **7**:
$E = 0.188$ eV, $\mathrm{RMSD_{min}} = 0.21$ Å, $|\vec{d}_{h\to e}| = 3.04$ Å

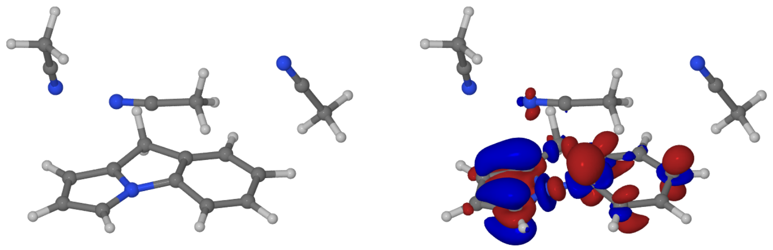

(g) cluster **8**:
$E = 0.195$ eV, $\mathrm{RMSD_{min}} = 0.15$ Å, $|\vec{d}_{h\to e}| = 3.06$ Å

S6 Molecular Geometries

In order to ensure that my results can be reproduced by other researchers, the present section lists the ground- and excited-state molecular geometries of fluorazene as optimized at the CAM-B3LYP and at the TD-CAM-B3LYP level of theory. All geometries are given in terms of Cartesian coordinates in units of ångström (Å).

The molecular geometries of fluorazene along the interpolated reaction path between S_1-LE minimum and the S_1-ICT (BQ) minimum, and the equilibrium geometries of the fluorazene−3 ACN clusters, are provided separately in ZIP files. Lastly, the simulated trajectories of the relaxation dynamics of fluorazene in the ACN nanodroplet have been made available at the Zenodo repository: https://doi.org/10.5281/zenodo.10966811

S_0-GS

Ground-state equilibrium geometry of fluorazene.

```
C    -0.366837    -0.270555     0.039015
N    -1.767598    -0.270978     0.007100
C    -2.272861     1.007860     0.097428
C    -3.642069     0.925779     0.055002
C    -3.964068    -0.463143    -0.065625
C    -2.787904    -1.178931    -0.093036
C     0.067819     1.059595     0.157163
C     1.424710     1.329395     0.209899
C     2.335138     0.268789     0.143854
C     1.884370    -1.043705     0.026541
C     0.518792    -1.334321    -0.027969
H    -4.346191     1.753264     0.103611
H    -4.963205    -0.890213    -0.125165
H    -2.605315    -2.247173    -0.174280
H     1.782986     2.357524     0.300997
H     2.606743    -1.861100    -0.023755
H     0.164934    -2.362201    -0.119640
H     3.406933     0.471942     0.184491
C    -1.134067     1.984660     0.204994
H    -1.163966     2.564526     1.141839
H    -1.122467     2.711455    -0.623453
```

S_1-LE

Locally excited minimum on the PES of state S_1.

C	-0.374533	-0.269442	0.038190
N	-1.748545	-0.274297	0.005484
C	-2.270171	1.024665	0.098351
C	-3.628208	0.928384	0.055938
C	-3.941258	-0.477297	-0.065379
C	-2.764181	-1.187525	-0.093460
C	0.046089	1.094550	0.159913
C	1.441082	1.359759	0.212832
C	2.323653	0.263287	0.143369
C	1.884551	-1.080835	0.023603
C	0.493306	-1.369275	-0.031864
H	-4.343299	1.745723	0.104057
H	-4.938657	-0.908633	-0.124479
H	-2.574945	-2.253887	-0.175211
H	1.825177	2.375815	0.302652
H	2.614764	-1.888097	-0.024951
H	0.117543	-2.388034	-0.123008
H	3.399031	0.452919	0.183099
C	-1.141632	2.008451	0.206921
H	-1.203159	2.605164	1.141481
H	-1.160726	2.751074	-0.618532

S_1-ICT (BQ)

Bent intramolecular charge transfer minimum on the PES of state S_1.

C	-1.171961	-1.434678	2.173557
N	-2.489299	-1.220054	1.575876
C	-2.808840	0.097727	1.523749
C	-4.140091	0.227425	0.992411
C	-4.572744	-1.044235	0.731142
C	-3.503071	-1.944680	1.113698
C	-0.603177	-0.099532	2.187627
C	0.757838	0.083254	2.150612
C	1.615898	-1.028935	2.042382
C	1.051604	-2.318702	1.855513
C	-0.306774	-2.534450	1.852829
H	-4.673930	1.161154	0.837362
H	-5.532057	-1.354093	0.323347
H	-3.469739	-3.030883	1.070671
H	1.176884	1.093523	2.182233
H	1.720843	-3.171098	1.709288
H	-0.700695	-3.543520	1.705830
H	2.697211	-0.894136	2.031946
C	-1.716396	0.924142	2.098572
H	-2.063922	1.298614	3.089054
H	-1.495781	1.817806	1.493459

S_1-TS

Transition state for interconversion between the S_1-LE and the S_1-ICT (BQ) structures.

C	-0.351985	-0.340393	0.358331
N	-1.756543	-0.319393	0.145896
C	-2.232374	0.988089	0.106112
C	-3.633663	0.947527	-0.124089
C	-3.972576	-0.382516	-0.216834
C	-2.758480	-1.158548	-0.047779
C	0.057805	1.052213	0.282777
C	1.407680	1.331213	0.111805
C	2.331887	0.297992	-0.010224
C	1.894237	-1.070825	-0.015494
C	0.562349	-1.386855	0.124966
H	-4.292908	1.808338	-0.192690
H	-4.961038	-0.808664	-0.374584
H	-2.626450	-2.237967	-0.063347
H	1.750241	2.369476	0.064681
H	2.629044	-1.870258	-0.127695
H	0.234382	-2.429473	0.090628
H	3.391383	0.525997	-0.133122
C	-1.143584	1.961509	0.379981
H	-1.314168	2.423899	1.388241
H	-1.159351	2.811106	-0.328553

References

[1] Yanai, T.; Tew, D. P.; Handy, N. C. A New Hybrid Exchange–Correlation Functional Using the Coulomb-Attenuating Method (CAM-B3LYP). *Chem. Phys. Lett.* **2004**, *393*, 51–57. DOI: 10.1016/j.cplett.2004.06.011

[2] Weigend, F.; Ahlrichs, R. Balanced Basis Sets of Split Valence, Triple Zeta Valence and Quadruple Zeta Valence Quality for H to Rn: Design and Assessment of Accuracy. *Phys. Chem. Chem. Phys.* **2005**, *7*, 3297–3305. DOI: 10.1039/b508541a

[3] Gaussian 16, Revision A.03, Frisch, M. J.; Trucks, G. W.; Schlegel, H. B.; Scuseria, G. E.; Robb, M. A.; Cheeseman, J. R.; Scalmani, G.; Barone, V.; Petersson, G. A.; Nakatsuji, H.; Li, X.; Caricato, M.; Marenich, A. V.; Bloino, J.; Janesko, B. G.; Gomperts, R.; Mennucci, B.; Hratchian, H. P.; Ortiz, J. V.; Izmaylov, A. F.; Sonnenberg, J. L.; Williams-Young, D.; Ding, F.; Lipparini, F.; Egidi, F.; Goings, J.; Peng, B.; Petrone, A.; Henderson, T.; Ranasinghe, D.; Zakrzewski, V. G.; Gao, J.; Rega, N.; Zheng, G.; Liang, W.; Hada, M.; Ehara, M.; Toyota, K.; Fukuda, R.; Hasegawa, J.; Ishida, M.; Nakajima, T.; Honda, Y.; Kitao, O.; Nakai, H.; Vreven, T.; Throssell, K.; Montgomery, J. A., Jr.; Peralta, J. E.; Ogliaro, F.; Bearpark, M. J.; Heyd, J. J.; Brothers, E. N.; Kudin, K. N.; Staroverov, V. N.; Keith, T. A.; Kobayashi, R.; Normand, J.; Raghavachari, K.; Rendell, A. P.; Burant, J. C.; Iyengar, S. S.; Tomasi, J.; Cossi, M.; Millam, J. M.; Klene, M.; Adamo, C.; Cammi, R.; Ochterski, J. W.; Martin, R. L.; Morokuma, K.; Farkas, O.; Foresman, J. B.; Fox, D. J. Gaussian, Inc., Wallingford CT, 2016.

[4] Pulay, P.; Fogarasi, G.; Pang, F.; Boggs, J. E. Systematic ab Initio Gradient Calculation of Molecular Geometries, Force Constants, and Dipole-Moment Derivatives. *J. Am. Chem. Soc.* **1979**, *101*, 2550–2560. DOI: 10.1021/ja00504a009

[5] Schlegel, H. B. Optimization of Geometries and Transition Structures. *J. Comput. Chem.* **1982**, *3*, 214–218. DOI: 10.1002/jcc.540030212

[6] Fogarasi, G.; Zhou, X.; Taylor, P. W.; Pulay, P. The Calculation of ab Initio Molecular Geometries: Efficient Optimization by Natural Internal Coordinates and Empirical Correction by Offset Forces. *J. Am. Chem. Soc.* **1992**, *114*, 8191–8201. DOI: 10.1021/ja00047a032

[7] Pulay, P.; Fogarasi, G. Geometry Optimization in Redundant Internal Coordinates. *J. Chem. Phys.* **1992**, *96*, 2856–2860. DOI: 10.1063/1.462844

[8] Baker, J. Techniques for Geometry Optimization: A Comparison of Cartesian and Natural Internal Coordinates. *J. Comput. Chem.* **1993**, *14*, 1085–1100. DOI: 10.1002/jcc.540140910

[9] Peng, C.; Schlegel, H. B. Combining Synchronous Transit and Quasi-Newton Methods for Finding Transition States. *Isr. J. Chem.* **1993**, *33*, 449–454. DOI: 10.1002/ijch.199300051

[10] Peng, C.; Ayala, P. Y.; Schlegel, H. B.; Frisch, M. J. Using Redundant Internal Coordinates to Optimize Equilibrium Geometries

and Transition States. *J. Comput. Chem.* **1996**, *17*, 49–56. DOI: 10.1002/(SICI)1096-987X(19960115)17:1<49::AID-JCC5>3.0.CO;2-0

[11] Li, X.; Frisch, M. J. Energy-Represented DIIS within a Hybrid Geometry Optimization Method. *J. Chem. Theory Comput.* **2006**, *2*, 835–839. DOI: 10.1021/ct050275a

[12] Plasser, A.; Thomitzni, B.; Bäppler, S. A.; Wenzel, J.; Rehn, D. R.; Wormit, W.; Dreuw, A. Statistical Analysis of Electronic Excitation Processes: Spatial Location, Compactness, Charge Transfer, and Electron-Hole Correlation. *J. Comp. Chem.* **2015**, *36*, 1609–1620. DOI: 10.1002/jcc.23975

[13] Grimme, S.; Parac, M. Substantial Errors from Time-Dependent Density Functional Theory for the Calculation of Excited States of Large π Systems. *ChemPhysChem* **2003**, *4*, 292–295. DOI: 10.1002/cphc.200390047

[14] Arulmozhiraja, S.; Coote, M. L. ^1L_a and ^1L_b States of Indole and Azaindole: Is Density Functional Theory Inadequate? *J. Chem. Theory Comput.* **2012**, *8*, 575–584. DOI: 10.1021/ct200768b

[15] Prlj, A.; Curchod, B. F. E.; Fabrizio, A.; Floryan, L.; Corminboeuf, C. Qualitatively Incorrect Features in the TDDFT Spectrum of Thiophene-Based Compounds. *J. Phys. Chem. Lett.* **2015**, *6*, 13–21. DOI: 10.1021/jz5022087

[16] Prlj, A.; Sandoval-Salinas, M. E.; Casanova, D.; Jacquemin, D.; Corminboeuf, C. Low-Lying $\pi\pi^*$ States of Heteroaromatic Molecules: A Challenge for Excited State Methods. J. Chem. Theory Comput. **2016**, *12*, 2652–2660. DOI: *10.1021/acs.jctc.6b00245*

[17] Acharya, A.; Chaudhuri, S.; Batista, V. S. Can TDDFT Describe Excited Electronic States of Naphthol Photoacids? A Closer Look with EOM-CCSD. *J. Chem. Theory Comput.* **2018**, *14*, 867–876. DOI: 10.1021/acs.jctc.7b01101

[18] Harbach, P. H. P.; Wormit, M.; Dreuw, A. The Third-Order Algebraic Diagrammatic Construction Method (ADC(3)) for the Polarization Propagator for Closed-Shell Molecules: Efficient Implementation and Benchmarking. *J. Chem. Phys.* **2014**, *141*, 064113. DOI: 10.1063/1.4892418

[19] Dreuw, A.; Wormit, M. The Algebraic Diagrammatic Construction Scheme for the Polarization Propagator for the Calculation of Excited States. *WIREs Comput. Mol. Sci.* **2015**, *5*, 82–95. DOI: 10.1002/wcms.1206

[20] Galván, I. F.; Martín, M. E.; Muñoz-Losa, A.; Aguilar, M. A. Dual Fluorescence of Fluorazene in Solution: A Computational Study. *J. Chem. Theory Comput.* **2011**, *7*, 3694–3701. DOI: 10.1021/ct2005227

[21] Xu, X.; Cao, Z.; Zhang, Q. Computational Characterization of Low-Lying States and Intramolecular Charge Transfers in *N*-Phenylpyrrole and the Planar-Rigidized Fluorazene. *J. Phys. Chem. A* **2006**, *110*, 1740–1748. DOI: 10.1021/jp055695a

[22] Roos, B. O. The Complete Active Space Self-Consistent Field Method and its Applications in Electronic Structure Calculations. In *Advances in Chemical Physics: Ab Initio Methods in Quantum Chemistry Part 2.* Lawley, K. P., Ed.; John Wiley & Sons Ltd., 1987; pp 399–445. DOI: 10.1002/9780470142943.ch7

[23] He, R.-X.; Li, X.-Y. An Anti-Quinoid Structure in Dual Fluorescence of Fluozazene Molecule and Solvent Effect of Intramolecular Charge Transfer. *Chem. Phys.* **2007**, *332*, 325–335. DOI: 10.1016/j.chemphys.2006.12.016

[24] Zilberg, S.; Haas, Y. The Nature of the Intramolecular Charge Transfer Excited State in *p*-Pyrrolocyanobenzene (PBN) and Other Derivatives of Benzene Substituted by Electron Donor and Acceptor Groups. *J. Phys. Chem. A* **2002**, *106*, 1-11. DOI: 10.1021/jp012573j

[25] Cogan, S.; Zilberg, S.; Haas, Y. The Electronic Origin of the Dual Fluorescence in Donor–Acceptor Substituted Benzene Derivatives. *J. Am. Chem. Soc.* **2006**, *128*, 3335–3345. DOI: 10.1021/ja0548945

[26] Yoshihara, T.; Druzhinin, S. I.; Zachariasse, K. A. Fast Intramolecular Charge Transfer with a Planar Rigidized Electron Donor/Acceptor Molecule. *J. Am. Chem. Soc.* **2004**, *126*, 8535–8539. DOI: 10.1021/ja049809s

[27] Druzhinin, S. I.; Kovalenko, S. A.; Senyushkina, T. A.; Demeter, A.; Zachariasse, K. A. Intramolecular Charge Transfer with Fluorazene and *N*-Phenylpyrrole. *J. Phys. Chem. A* **2010**, 114, 1621–1632. DOI: 10.1021/jp909682p

[28] Pabst, M.; Köhn, A. Implementation of Transition Moments between Excited States in the Approximate Coupled-Cluster Singles and Doubles Model. *J. Chem. Phys.* **2008**, *129*, 214101. DOI: 10.1063/1.3023118

[29] Shiozaki, T.; Győrffy, W.; Celani, P.; Werner, H.-J. Communication: Extended Multi-State Complete Active Space Second-Order Perturbation Theory: Energy and Nuclear Gradients. *J. Chem. Phys.* **2011**, *135*, 081106. DOI: 10.1063/1.3633329

[30] Park, J. W.; Shiozaki, T. Analytical Derivative Coupling for Multistate CASPT2 Theory. *J. Chem. Theory Comput.* **2017**, *13*, 2561–2570. DOI: 10.1021/acs.jctc.7b00018

[31] BAGEL, Brilliantly Advanced General Electronic-structure Library. http://www.nubakery.org under the GNU General Public License.

[32] Shiozaki, T. BAGEL: Brilliantly Advanced General Electronic-structure Library. *Wiley Interdiscip. Rev.: Comput. Mol. Sci.* **2017**, e1331. DOI: 10.1002/wcms.1331

[33] Finley J.; Malmqvist, P.-Å.; Roos, B. O.; Serrano-Andrés, L. The Multi-State CASPT2 Method. *Chem. Phys. Lett.* **1998**, *288*, 299–306. DOI: 10.1016/S0009-2614(98)00252-8

[34] Dunning Jr, T. H. Gaussian Basis Sets for Use in Correlated Molecular Calculations. I. The Atoms Boron Through Neon and Hydrogen. *J. Chem. Phys.* **1989**, *90*, 1007–1023. DOI: 10.1063/1.456153

[35] Weigend, F. A Fully Direct RI-HF Algorithm: Implementation, Optimised Auxiliary Basis Sets, Demonstration of Accuracy and Efficiency. *Phys. Chem. Chem. Phys.* **2002**, *4*, 4285–4291. DOI: 10.1039/B204199P

[36] Correlation Consistent J&K-fit Density Fitting Basis Sets Consistent with Molpro 2009. https://github.com/psi4/psi4/blob/master/psi4/share/psi4/basis/aug-cc-pvdz-jkfit.gbs, accessed on May 16, 2024.

[37] Senn, H. M.; Thiel, W. QM/MM Methods for Biomolecular Systems. *Angew. Chem. Int. Ed.* **2009**, *48*, 1198–1229. DOI: 10.1002/anie.200802019

[38] Groenhof, G. Introduction to QM/MM Simulations. In *Biomolecular Simulations. Methods in Molecular Biology, vol. 924.* Monticelli, L.; Salonen, E., Eds.; Humana Press, 2013. DOI: 10.1007/978-1-62703-017-5_3

[39] Mennucci, B.; Corni, S. Multiscale Modelling of Photoinduced Processes in Composite Systems. *Nat. Rev. Chem.* **2019**, *3*, 315–330. DOI: 10.1038/s41570-019-0092-4

[40] Nottoli, M.; Cupellini, L.; Lipparini, F.; Granucci, G; Mennucci, B. Multiscale Models for Light-Driven Processes. *Annu. Rev. Phys. Chem.* **2021**, *72*, 1545–1593. DOI: 10.1146/annurev-physchem-090419-104031

[41] Toldo, J. M.; do Casal, M. T.; Ventura, E.; do Monte, S. A.; Barbatti, M. Surface Hopping Modeling of Charge and Energy Transfer in Active Environments. *Phys. Chem. Chem. Phys.* **2023**, *25*, 8293–8316. DOI: 10.1039/D3CP00247K

[42] Schweke, D.; Haas, Y. The Fluorescence of *N*-Phenylpyrrole in an Argon/Acetonitrile Matrix. *J. Phys. Chem. A* **2003**, *107*, 9554–9560. DOI: 10.1021/jp035959y

[43] Jorgensen, W. L.; Maxwell, D. S.; Tirado-Rives, J. Development and Testing of the OPLS All-Atom Force Field on Conformational Energetics and Properties of Organic Liquids. *J. Am. Chem. Soc.* **1996**, *118*, 11225–11236. DOI: 10.1021/ja9621760

[44] Price, M. L. P.; Ostrovsky, D.; Jorgensen, W. L. Gas-Phase and Liquid-State Properties of Esters, Nitriles, and Nitro Compounds with the OPLS-AA Force Field. *J. Comput. Chem.* **2001**, *22*, 1340–1352. DOI: 10.1002/jcc.1092

[45] Ruckenbauer, M.; Barbatti, M.; Müller, T.; Lischka, H. Nonadiabatic Photodynamics of a Retinal Model in Polar and Nonpolar Environment. *J. Phys. Chem. A* **2013**, *117*, 2790–2799. DOI: 0.1021/jp400401f

[46] Avagliano, D.; Lorini, E.; González, L. Sampling Effects in Quantum Mechanical/Molecular Mechanics Trajectory Surface Hopping Non-Adiabatic Dynamics. *Phil. Trans. R. Soc. A.* **2022**, *380*, 20200381. DOI: 10.1098/rsta.2020.0381

[47] Bowman, J. M.; Gazdy, B.; Sun, Q. A Method to Constrain Vibrational Energy in Quasiclassical Trajectory Calculations. *J. Chem. Phys.* **1989**, *91*, 2859–2862. DOI: 10.1063/1.456955

[48] Guo, Y.; Thompson, D. L.; Sewell, T. D. Analysis of the Zero-Point Energy Problem in Classical Trajectory Simulations. *J. Chem. Phys.* **1996**, *104*, 576–582. DOI: 10.1063/1.470853

[49] Mukherjee, S.; Barbatti, M. A Hessian-Free Method to Prevent Zero-Point Energy Leakage in Classical Trajectories. *J. Chem. Theory Comput.* **2022**, *18*, 4109–4116. DOI: 10.1021/acs.jctc.2c00216

[50] Singh, U. C.; Kollman, P. A. An Approach to Computing Electrostatic Charges for Molecules. *J. Comp. Chem.* **1984**, *5*, 129–145. DOI: 10.1002/jcc.540050204

[51] Brünger, A.; Brooks III, C. L.; Karplus, M. Stochastic Boundary Conditions for Molecular Dynamics Simulations of ST2 Water. *Chem. Phys. Lett.* **1984**, *105*, 495–500. DOI: 10.1016/0009-2614(84)80098-6

[52] Barbatti, M.; Aquino, A. J. A.; Lischka, H. The UV absorption of Nucleobases: Semi-Classical *Ab Initio* Spectra Simulations. *Phys. Chem. Chem. Phys.* **2010**, *12*, 4959-4967. DOI: 10.1039/B924956G

[53] Crespo-Otero, R.; Barbatti, M. Spectrum Simulation and Decomposition with Nuclear Ensemble: Formal Derivation and Application to Benzene, Furan and 2-Phenylfuran. *Theor. Chem. Acc.* **2012**, *131*, 1237. DOI: 10.1007/s00214-012-1237-4

[54] Tully, J. C.; Preston, R. K. Trajectory Surface Hopping Approach to Nonadiabatic Molecular Collisions: The Reaction of H^+ with D_2. *J. Chem. Phys.* **1971**, *55*, 562–572. DOI: 10.1063/1.1675788

[55] Tully, J. C. Molecular Dynamics with Electronic Transitions. *J. Chem. Phys.* **1990**, *93*, 1061–1071. DOI: 10.1063/1.459170

[56] Hammes-Schiffer, S.; Tully, J. C. Proton Transfer in Solution: Molecular Dynamics With Quantum Transitions. *J. Chem. Phys.* **1994**, *101*, 4657–4667. DOI: 10.1063/1.467455

[57] Granucci, G.; Persico, M. Critical Appraisal of the Fewest Switches Algorithm for Surface Hopping. *J. Chem. Phys.* **2007**, *126*, 134114. DOI: 10.1063/1.2715585

[58] Zhang, X.; Herbert, J. M. Analytic Derivative Couplings in Time-Dependent Density Functional Theory: Quadratic Response Theory versus Pseudo-Wavefunction Approach. *J. Chem. Phys.* **2015**, *142*, 064109. DOI: 10.1063/1.4907376

[59] Bil, A.; Kochman, M. A. Photoinduced Double Proton Transfer in the Glyoxal–Methanol Complex Revisited: The Role of the Excited States. *J. Chem. Theory Comput.* **2020**, *16*, 3273–3286. DOI: 10.1021/acs.jctc.0c00007

[60] Kochman, M. A.; Gryber, T.; Durbeej, B.; Kubas, A. Simulation and Analysis of the Relaxation Dynamics of a Photochromic Furylfulgide. *Phys. Chem. Chem. Phys.* **2022**, *24*, 18103–18118. DOI: 10.1039/D2CP02143A

[61] Kochman, M. A.; Durbeej, B. Simulating the Nonadiabatic Relaxation Dynamics of 4-(*N*,*N*-Dimethylamino)benzonitrile (DMABN) in Polar Solution. *J. Phys. Chem. A* **2020**, *124*, 2193–2206. DOI: 10.1021/acs.jpca.9b10588

[62] Kochman, M. A.; Durbeej, B.; Kubas, A. Simulation and Analysis of the Transient Absorption Spectrum of 4-(*N*,*N*-Dimethylamino)benzonitrile (DMABN) in Acetonitrile *J. Phys. Chem. A* **2021**, 125, 8635–8648. DOI: 10.1021/acs.jpca.1c06166

[63] Barbatti, M. Photorelaxation Induced by Water–Chromophore Electron Transfer. *J. Am. Chem. Soc.* **2014**, *136*, 10246–10249. DOI: 10.1021/ja505387c

[64] Barbatti, M.; Lischka, H. Why Water Makes 2-Aminopurine Fluorescent? *Phys. Chem. Chem. Phys.* **2015**, *17*, 15452–15459. DOI: 10.1039/C5CP01151E

[65] Szabla, R.; Kruse, H.; Šponer, J.; Góra, R. W. Electron-Driven Proton Transfer along H_2O Wires Enables Photorelaxation of $\pi\sigma^*$ States in Chromophore–Water Clusters. *J. Phys. Chem. Lett.* **2015**, 6, 1467–1471. DOI: 10.1021/acs.jpclett.5b00261

[66] Szabla, R.; Góra, R. W.; Janicki, M.; Šponer, J. Photorelaxation of Imidazole and Adenine via Electron-Driven Proton Transfer along H_2O Wires. *Faraday Discuss.* **2016**, *195*, 237–251. DOI: 10.1039/C6FD00131A

[67] Szabla, R.; Kruse, H.; Šponer, J.; Góra, R. W. Water–Chromophore Electron Transfer Determines the Photochemistry of Cytosine and Cytidine. *Phys. Chem. Chem. Phys.* **2017**, *19*, 17531–17537. DOI: 10.1039/C7CP02635H

[68] Gómez, S.; Soysal, E. N.; Worth, G. A. Micro-Solvated DMABN: Excited State Quantum Dynamics and Dual Fluorescence Spectra. *Molecules* **2021**, *26*, 7247. DOI: 10.3390/molecules26237247

[69] Grimme, S.; Ehrlich, S.; Goerigk, L. Effect of the Damping Function in Dispersion Corrected Density Functional Theory. *J. Comp. Chem.* **2011**, *32*, 1456–1465. DOI: 10.1002/jcc.21759

[70] Liu, J.; Liang, W. Analytical Hessian of Eelectronic Excited States in Time-Dependent Density Functional Theory with Tamm-Dancoff Approximation. *J. Chem. Phys.* **2011**, *135*, 014113. DOI: 10.1063/1.3605504

[71] Liu, J.; Liang, W. Analytical Approach for the Excited-State Hessian in Time-Dependent Density Functional Theory: Formalism, Implementation, and Performance. *J. Chem. Phys.* **2011**, *135*, 184111. DOI: 10.1063/1.3659312
